# Bridging the gap between movement data and connectivity analysis using the Time-Explicit Habitat Selection (TEHS) model

**DOI:** 10.1186/s40462-024-00461-1

**Published:** 2024-03-01

**Authors:** Denis Valle, Nina Attias, Joshua A. Cullen, Mevin B. Hooten, Aline Giroux, Luiz Gustavo R. Oliveira-Santos, Arnaud L. J. Desbiez, Robert J. Fletcher

**Affiliations:** 1 School of Forest, Fisheries, and Geomatics Sciences, University of Florida, Gainesville, FL USA; 2 Instituto de Conservação de Animais Silvestres, Campo Grande, Mato Grosso do Sul Brazil; 3 Department of Earth, Ocean, and Atmospheric Science, Florida State University, Tallahassee, FL USA; 4 Department of Statistics and Data Sciences, University of Texas at Austin, Austin, TX USA; 5 Ecology Department, Federal University of Mato Grosso do Sul, Campo Grande, Mato Grosso do Sul Brazil; 6 Royal Zoological Society of Scotland, Murrayfield, Edinburgh, UK; 7 Instituto de Pesquisas Ecologicas, Nazare Paulista, Sao Paulo Brazil; 8 Department of Wildlife Ecology and Conservation, University of Florida, P.O. Box 110410, Gainesville, FL USA

**Keywords:** Connectivity analysis, Landscape resistance, Step selection, Giant anteater, Landscape use, Movement ecology, Wildlife corridors

## Abstract

**Background:**

Understanding how to connect habitat remnants to facilitate the movement of species is a critical task in an increasingly fragmented world impacted by human activities. The identification of dispersal routes and corridors through connectivity analysis requires measures of landscape resistance but there has been no consensus on how to calculate resistance from habitat characteristics, potentially leading to very different connectivity outcomes.

**Methods:**

We propose a new model, called the Time-Explicit Habitat Selection (TEHS) model, that can be directly used for connectivity analysis. The TEHS model decomposes the movement process in a principled approach into a time and a selection component, providing complementary information regarding space use by separately assessing the drivers of time to traverse the landscape and the drivers of habitat selection. These models are illustrated using GPS-tracking data from giant anteaters (*Myrmecophaga tridactyla*) in the Pantanal wetlands of Brazil.

**Results:**

The time model revealed that the fastest movements tended to occur between 8 p.m. and 5 a.m., suggesting a crepuscular/nocturnal behavior. Giant anteaters moved faster over wetlands while moving much slower over forests and savannas, in comparison to grasslands. We also found that wetlands were consistently avoided whereas forest and savannas tended to be selected. Importantly, this model revealed that selection for forest increased with temperature, suggesting that forests may act as important thermal shelters when temperatures are high. Finally, using the spatial absorbing Markov chain framework, we show that the TEHS model results can be used to simulate movement and connectivity within a fragmented landscape, revealing that giant anteaters will often not use the shortest-distance path to the destination patch due to avoidance of certain habitats.

**Conclusions:**

The proposed approach can be used to characterize how landscape features are perceived by individuals through the decomposition of movement patterns into a time and a habitat selection component. Additionally, this framework can help bridge the gap between movement-based models and connectivity analysis, enabling the generation of time-explicit connectivity results.

**Supplementary Information:**

The online version contains supplementary material available at 10.1186/s40462-024-00461-1.

## Background

Land-use change is the major driver of biodiversity loss in terrestrial and freshwater ecosystems across the world [11] and the resulting habitat loss and fragmentation has been a central theme for conservation biologists [22, 24], particularly regarding how to connect habitat remnants to facilitate the movement of wildlife [7, 18]. Identifying wildlife dispersal routes and potential corridors through connectivity analysis typically requires the quantification of landscape resistance [15, 23, 55] and this is often measured as a function of proxies of habitat quality, such as the estimated presence probability derived from species distribution models (e.g., [35]) or derived from habitat selection models (e.g., [56]). However, connectivity analyses typically use arbitrary equations to transform these proxies of habitat quality into resistance (but see [25, 50]). For example, resistance has been often assumed to be the inverse of the predicted probability of presence [56], but other transformations have also been applied (e.g., [29, 34, 35]). In contrast to focusing on resistance, some authors have argued that wildlife corridors should be based on areas in which animals move faster and in a directed fashion (i.e., exhibit transit behavior) [2, 34] instead of areas with higher habitat quality.

Recent work from Hofmann et al. [25] has combined habitat selection and speed to generate connectivity maps while avoiding these arbitrary transformations to calculate landscape resistance. In the first step of their analysis, animal movement data is analyzed using the integrated Step Selection Analysis (iSSA; [4]), an approach that accounts for how habitat characteristics influence both speed and selection processes. Then, an individual-based movement model is used to simulate potential trajectories based on the estimated parameters of iSSA. Finally, these trajectories are summarized into various connectivity maps. In this article, we propose an alternative approach that also builds on the idea that time taken to traverse a particular path (or its reciprocal, speed) and habitat selection strength are distinct axes that together can help improve understanding of dispersal and connectivity across the landscape (Box 1) (see also [12, 32]). More specifically, we develop a novel habitat selection model that decomposes movement in these two processes, enabling a better understanding of resource selection. This model generates a probabilistic metric of habitat selection that can be used in connectivity analysis without requiring arbitrary transformations. We provide an example by modeling empirical movement data of giant anteaters (*Myrmecophaga tridactyla*) in the floodable savannas of Brazil.

### Box 1: Conceptual framework

**Fig. 1:**
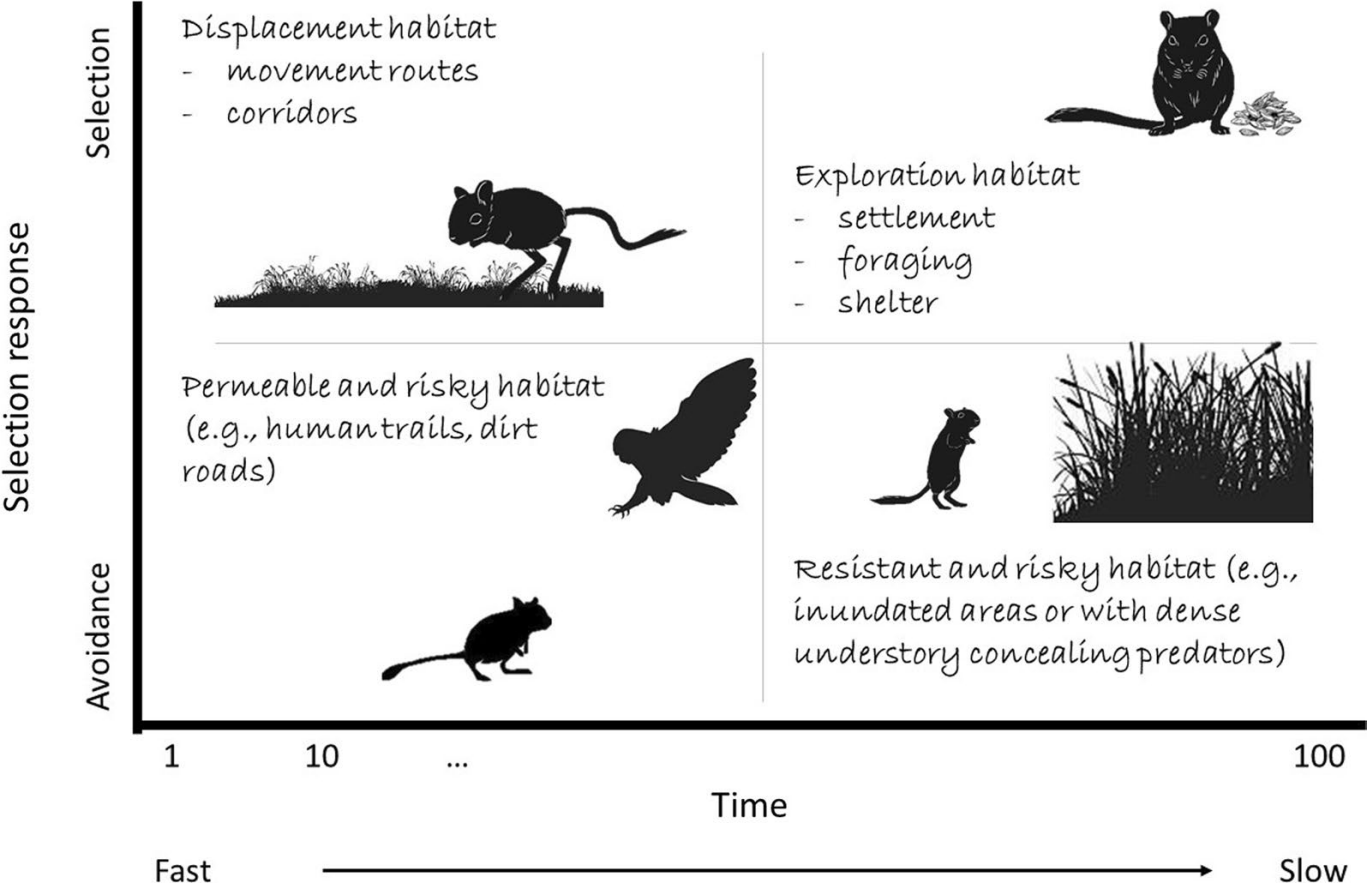
Time to traverse the landscape and selection strength are two important axes for characterizing the landscape from the perspective of a species. The variable in the x-axis is time taken to traverse a particular distance and, as a result, shorter or longer periods of time correspond to faster and slower movements, respectively

It is important to take into account both selection strength and time to traverse the landscape. Relying just on selection strength, while ignoring time, limits the understanding of animal resource use. For example, a selected habitat might be selected for displacement (often resulting in faster movements and shorter time in that area; upper left quadrant in Fig. 1) or for resource use, such as foraging and shelter (often resulting in slower movements and longer time in the area; upper right quadrant in Fig. 1; [3, 57]). Similarly, a habitat type might be avoided because it presents a high mortality risk (often resulting in faster movements and shorter time; lower left quadrant in Fig. 1) or because it is a physical barrier to movement (often resulting in slower movements and longer time; lower right quadrant in Fig. 1; [5, 12, 47]).

Only accounting for the time taken to traverse the landscape while ignoring selection strength can also be problematic [12]. For example, residence time (i.e., the time an individual spends in a given area once it is reached) has been used as an indicator of high-quality resource areas used by animals (e.g., herbivore foraging patches or carnivore kill site; [49, 51]). However, increased time within a particular type of landscape could simply indicate the presence of greater biomechanical resistance to movement (e.g., due to greater number of obstacles/barriers or steeper slopes) [3]. Time taken to traverse a landscape can also be related to perceptual range (e.g., visually oriented small mammals tend to increase their perceptual range in habitats with low vegetation height, which allows for faster and more directed movement; [46]) and memory (e.g., prior knowledge of resource location can result in faster and more directed movement towards a given type of habitat; [14, 42]).

Examining time and selection strength as separate axes of movement can help distinguish between different motivations for movement. For example, we illustrate in Fig. 1 how fast movement and selection can characterize a displacement habitat (upper left quadrant) whereas slow movement and selection might be the hallmark of resource exploration habitat (upper right quadrant). Similarly, fast movement associated with avoidance might indicate permeable but risky habitat (lower left quadrant) whereas slow movement and avoidance suggest resistant and risky habitat (lower right quadrant).

In short, selection strength and time required to traverse the landscape can provide complementary insights to determine whether a landscape characteristic is perceived as a movement corridor, a source of foraging and shelter, or a source of risk, with important implications for connectivity.

## Material and methods

### Linking habitat selection models with connectivity analysis

Most of the prominent methods used for connectivity analysis rely on estimates of cost of movement in the form of resistance surfaces [15, 33]. In this article, however, we focus on parameterizing a permeability/conductance matrix (instead of a resistance surface) because this enables us to decompose movement patterns into a time component and a habitat selection component.

Each cell in the permeability matrix contains the probability of choosing a particular pixel *j* given time constraint ${\Delta }t$ and initial pixel *i* (i.e., $p\left( {P_{t + \Delta t} = j|\Delta t,P_{t} = i} \right)$). Using Bayes theorem, this probability is given by:

1 $$p\left( {P_{t + \Delta t} = j|\Delta t,P_{t} = i} \right) = \frac{{p\left( {\Delta t|P_{t + \Delta t} = j,P_{t} = i} \right)p\left( {P_{t + \Delta t} = j|P_{t} = i} \right)}}{{\mathop \sum \nolimits_{k = 1}^{N} p\left( {\Delta t|P_{t + \Delta t} = k,P_{t} = i} \right)p\left( {P_{t + \Delta t} = k|P_{t} = i} \right)}},$$

where *N* is the number of pixels in the landscape $\left( {{\text{i}}{{\rm .e}}{.},\; P_{t + \Delta t} \in \left\{ {1, \ldots ,N} \right\}} \right)$. Note that the time component $p\left( {\Delta t|P_{t + \Delta t} = j,P_{t} = i} \right)$ quantifies the likelihood that ${\Delta }t$ will be required to reach pixel $P_{t + \Delta t} = j$ from pixel $P_{t} = i$, whereas the selection component $p\left( {P_{{t + {\Delta }t}} = j{|}P_{t} = i} \right)$ quantifies selection strength for pixel *j* given initial pixel *i* regardless of time constraints. This expression is similar to the equations commonly used for habitat selection models. For example, if $p\left( {{\Delta }t{|}P_{{t + {\Delta }t}} = j,P_{t} = i} \right)$ is a constant for all *N* pixels, then this quantity cancels out in the numerator and denominator and this expression becomes identical to those used in standard step selection (SSF) and resource selection (RSF) functions. In short, Eq. 1 decomposes the movement process into a time and a selection component. Because this decomposition relies on a well-accepted mathematical relationship (i.e., Bayes theorem), this model formulation does not require the commonly adopted assumption in SSFs of separable movement and habitat selection kernels.

Permeability matrices are a key part of popular connectivity models, such as circuit theoretic connectivity analysis [38] and the spatial absorbing Markov chain (SAMC) framework [17]. In this article, we rely on SAMC to link habitat selection models to connectivity analysis. This framework is based on random walk theory and captures the initiation and termination of movement, how the environment alters movement behavior, and how these processes can impact demographic rates. Aside from the permeability matrix (**Q)**, SAMC may also require information on the initial distribution of a species (${{\varvec{\Psi}}})$ and information on factors that may terminate movement (e.g., mortality risk from roads and settlement) (${\mathbf{R}})$. Depending on the application, all or only subsets of these components might need to be considered. Importantly, unlike other common connectivity models such as least-cost analysis or circuit-theoretic models [13, 38], SAMC can provide time-explicit results in addition to long-term analytical solutions for multiple connectivity metrics.

The time-explicit nature of SAMC allows us to directly relate our movement model results to landscape connectivity. More specifically, we develop a model to explicitly estimate $p\left( {{\Delta }t{|}P_{{t + {\Delta }t}} = j,P_{t} = i} \right)$ in Eq. 1 (onwards simply “time model”) by assuming that ${\Delta }t$ is the time interval between GPS fixes. The results from this model are then used together with the selection function $\left( {P_{{t + {\Delta }t}} = j{|}P_{t} = i} \right)$, yielding the Time-Explicit Habitat Selection (TEHS) model. As described in Box 1, this decomposition of movement into a time component and a selection component can improve the understanding of how animals use the landscape and disperse to new areas. Once the parameters from the time model and the selection function have been estimated, they can be used to calculate the movement probabilities that are part of the transition matrix **Q** (i.e., $q_{ij} = p\left( {P_{t + \Delta t} = j|\Delta t,P_{t} = i} \right)$) in SAMC and the permeability matrix of other connectivity models. Below, we start by first describing the time model to then describe the habitat selection component within the TEHS model.

### Time model

**Fig. 2:**
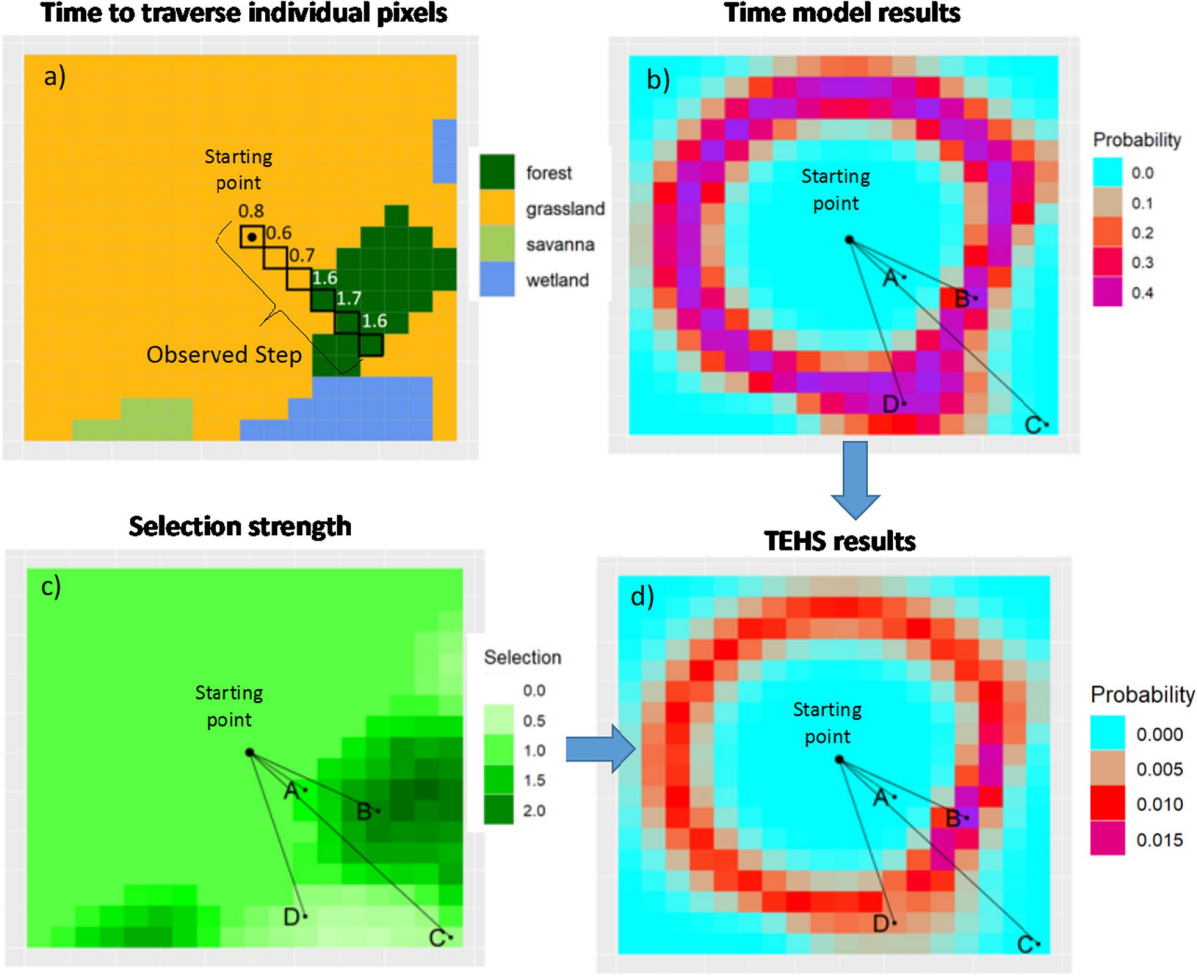
Conceptual description of the time model and habitat selection function within the TEHS model. **a** A hypothetical landscape illustrating the time taken to traverse a particular path. Traversed pixels are shown with black squares and the time (in min) taken to traverse each pixel is shown above the corresponding pixel. **b** Time model results regarding the probability of taking 7 min to reach each pixel of the landscape given the initial starting point. **c** Selection strength for the different landscape characteristics, where values > 1 indicate selection and values < 1 indicate avoidance relative to grassland. **d** Movement probabilities based on the TEHS model once the results of the time model and selection strength are taken into account. In panels **b**–**d** we show four potential endpoints for the step (points A–D) in this landscape

We illustrate the main concepts underlying the time model using a simple hypothetical example (Fig. 2). Figure 2a depicts the path taken by a hypothetical individual in a given step (i.e., the path defined by two consecutive GPS fixes) where step length is 6 pixels and the animal takes 7 min overall to traverse these pixels. The first three pixels are comprised of grasslands and the animal takes 0.8, 0.6 and 0.7 min to traverse these pixels whereas the next three pixels are traversed much more slowly (i.e., 1.6, 1.7, and 1.6 min per pixel) because they are forested pixels. Importantly, only the time taken to traverse all 6 pixels is known when using location data (i.e., the time taken to traverse individual pixels is latent and therefore must be estimated).

We start by assuming that the time taken to traverse pixel *i* in step *j* (${\Delta }t_{ij}$) is given by

$${\Delta }t_{ij} \sim {\text{Gamma}}\left( {a_{ij} ,b} \right),$$

where we assume that $E\left[ {{\Delta }t_{ij} } \right] = \frac{{a_{ij} }}{b} = D_{ij} \exp \left( {{\varvec{x}}_{{\varvec{i}}}^{T} {\varvec{\beta}}} \right)$. In this expression, $D_{ij}$ is the distance traveled in pixel *i* in step *j*, ${\varvec{x}}_{{\varvec{i}}}^{T}$ is a vector of covariates associated with pixel *i*, and ${\varvec{\beta}}$ is a vector of regression coefficients. In this gamma regression, time taken to traverse a pixel is assumed to be proportional to the distance traveled but the proportionality constant depends on the characteristics of the pixel. As a result, these slope parameters determine how the mean time taken to traverse a pixel is associated with pixel-level variables such as land-use/land-cover (LULC), elevation, distance to road, or normalized difference vegetation index (NDVI).

Let ${\Delta }t_{.j} = \sum\nolimits_{i = 1}^{{n_{j} }} {{\Delta }t_{ij} }$ be the total amount of time in step *j*, where $n_{j}$ is the number of pixels traversed within step *j*. Notice that using standard GPS tracking data, we only observe ${\Delta }t_{.j}$ while the individual times for each pixel ${\Delta }t_{ij}$ are latent. For example, in Fig. 2a, the total time taken to traverse these 6 pixels was equal to 7 min, but we do not know the time required to cross each individual pixel. Nevertheless, because of the Gamma distribution assumption, it can be shown that

$${\Delta }t_{.j} \sim {\text{Gamma}}\left( {\mathop \sum \limits_{i = 1}^{{n_{j} }} a_{ij} ,b} \right).$$

This model can be viewed as representing a gamma process in which, because individual increments arise from gamma distributions, the sum of increments is also gamma distributed. Unfortunately, it can be cumbersome to repeatedly calculate $E\left[ {{\Delta }t_{.j} } \right] = \frac{{\mathop \sum \nolimits_{i = 1}^{{n_{j} }} a_{ij} }}{b} = \sum\nolimits_{i = 1}^{{n_{j} }} {D_{ij} \exp \left( {{\varvec{x}}_{{\varvec{i}}}^{T} {\varvec{\beta}}} \right)}$ within our model fitting algorithm. To enable this model to be fit in a straightforward fashion, we approximate this quantity by using the mean of the covariates values for the pixels traversed in step *j* ($\overline{\user2{x}}_{{\varvec{j}}}^{T}$) as well as the overall distance in this step ($D_{j}$):

$$\mathop \sum \limits_{i = 1}^{{n_{j} }} D_{ij} \exp \left( {{\varvec{x}}_{{\varvec{i}}}^{T} {\varvec{\beta}}} \right) \approx D_{j} \exp \left( {\overline{\user2{x}}_{{\varvec{j}}}^{T} {\varvec{\beta}}} \right)$$.

The accuracy of this approximation is likely to be higher if the pixels that characterize the environment are large relative to the step lengths and/or if there is little spatial heterogeneity in the landscape. We test this approximation using simulated data and find that our model works well (see Additional file 1: Appendix 1). As a result of this approximation, our model becomes ${\Delta }t_{.j} \sim {\text{Gamma}}\left( {bD_{j} \exp \left( {\overline{\user2{x}}_{{\varvec{j}}}^{T} {\varvec{\beta}}} \right),b} \right).$

An extension of this basic model allows us to account for additional variability associated with missed fixes. Although tracking devices are often programmed to collect GPS coordinates at regular time intervals, ${\Delta }t_{.j}$ can be substantially different from the programmed time interval due to missing GPS fixes. When GPS fixes are missed, it is likely that there will be even greater uncertainty regarding the exact path traveled by the animal. For this reason, we modify the above model to allow the variance to potentially increase when GPS fixes are missed

$${\Delta }t_{.j} \sim {\text{Gamma}}\left( {\exp \left( {\gamma_{0} + \gamma_{1} M_{j} } \right)D_{j} \exp \left( {\overline{\user2{x}}_{{\varvec{j}}}^{T} {\varvec{\beta}}} \right),\exp \left( {\gamma_{0} + \gamma_{1} M_{j} } \right)} \right),$$

where $M_{j}$ is a binary variable, equal to 1 if GPS fixes were missing in step *j* and equal to zero otherwise. In this expression, the variance is given by $Var\left[ {{\Delta }t_{.j} } \right] = \frac{{D_{j} \exp \left( {\overline{\user2{x}}_{{\varvec{j}}}^{T} {\varvec{\beta}}} \right)}}{{\exp \left( {\gamma_{0} + \gamma_{1} M_{j} } \right)}}$. If missed GPS fixes increase the variance, we expect that $\gamma_{1} < 0$ because the denominator will be smaller when $M_{j}$ = 1 and therefore the variance will be larger.

Besides providing inference on how landscape variables (e.g., land use) influence time to traverse a pixel, this model also estimates the probability of reaching different parts of the landscape in a particular time interval, assuming an initial location. For example, Fig. 2b displays the probability that the animal requires 7 min to reach each pixel in the landscape assuming the animal starts at the center black dot and moves in a straight line. Areas close to the starting point (e.g., point A in Fig. 2b) have low probability because much less than 7 min are needed to reach these pixels. On the other hand, point C in Fig. 2b also has low probability because much more than 7 min is required to reach this pixel given that it is very far away from the starting point. Notice that Fig. 2b is asymmetric because, in this example, time taken to traverse the landscape is influenced by the LULC classes along the path. For instance, the probability of taking 7 min to reach point B is similar to that of reaching point D, despite the fact that point B is closer to the starting point when compared to D. This occurs because the animal moves faster over the path required to reach D (due to the lower proportion of forests and presence of wetlands) whereas the animal moves slower over the path required to reach B (due to the higher proportion of forests and absence of wetlands).

### Habitat selection function within TEHS

To illustrate how the Time-Explicit Habitat Selection (TEHS) model works, it is useful to refer back to Fig. 2. Figure 2c reveals that the animal might have moved from the starting point to point B but other steps would also have been possible (e.g., the path from the starting point to point D). Irrespective of its movement capability, in this example, individuals tend to select forests and savanna in relation to grassland while avoiding wetlands. Figure 2d shows that the step ultimately chosen by the animal is driven by a combination of the likelihood of the animal reaching that pixel within a given time interval (estimated by the time model) and selection strength for that path irrespective of movement constraints (habitat selection function).

To more clearly explain the TEHS model, assume that the fix rate from our GPS tracking device is ${\Delta }t$, that the animal is currently in pixel *i* (i.e., $P_{t} = i$), and that the landscape contains *N* pixels $\left( {{\text{i}}.{\text{e}}., \;P_{{t + {\Delta }t}} \in \left\{ {1, \ldots ,N} \right\}} \right)$. Furthermore, recall that we are interested in estimating the probability of choosing pixel *j* given time constraint ${\Delta }t$ and starting point *i* (i.e., $q_{ij} = p\left( {P_{t + \Delta t} = j|\Delta t,P_{t} = i} \right)$) (Eq. 1). In this equation, $p\left( {{\Delta }t{|}P_{{t + {\Delta }t}} = j,P_{t} = i} \right)$ quantifies the likelihood that ${\Delta }t$ will be required to traverse the path between $P_{t} = i$ and $P_{{t + {\Delta }t}} = j$ and can be calculated based on the time model described previously.

We assume that the habitat selection model (i.e., $p\left( {P_{{t + {\Delta }t}} = j{|}P_{t} = i} \right)$ in Eq. 1) is given by $\frac{{\exp \left( {\overline{\user2{x}}_{{{\varvec{ij}}}}^{T} {\varvec{\alpha}}} \right)}}{{\mathop \sum \nolimits_{k}^{N} \exp \left( {\overline{\user2{x}}_{{{\varvec{ij}}}}^{T} {\varvec{\alpha}}} \right)}}$, where $\overline{\user2{x}}_{{{\varvec{ij}}}}^{T}$ is a vector that contains the mean covariate values in the path from *i* to *j* and ${\varvec{\alpha}}$ is a vector containing the corresponding slope parameters. We rely on average covariate values in order to be consistent with the time model formulation, but this is not required (i.e., just the covariate values at the destination pixel *j* could have been used). Our model calculates the probability of moving from pixel *i* to pixel *j* by first specifying the marginal habitat-selection probability and multiplying it by the conditional time probability. This conditional time probability, given by the time model, will automatically distinguish pixels that are available from ones that are not. For instance, a pixel that is too far away for the animal to reach within a particular time interval ${\Delta }t$ will be essentially removed from Eq. 1 because $p\left( {{\Delta }t{|}P_{{t + {\Delta }t}} = j,P_{t} = i} \right) \approx 0$.

To fit the habitat selection model, we start by noting that the denominator in Eq. 1 is similar to that in SSF models, except that the integral is replaced by a sum because we are assuming discrete (rather than continuous) space. As in SSF models, it can be computationally expensive to calculate this denominator because, in the case of Eq. 1, the sum is over all potential destination pixels in the study area (i.e., *N* might be very large) (e.g., [1, 39, 44]). Various approaches to calculate the SSF denominator are reported in the literature, including Monte Carlo with known movement kernel, uniform Monte Carlo, importance sampling, and uniform quadrature [39].

Instead of attempting to approximate these denominators, we rely on the approach described in Manly et al. [, chapter 8] for SSFs. Assuming space is discrete, we write our likelihood as:^36^

2 $${\varvec{y}}_{{{\varvec{t}} + 1}} \sim {\text{Multinom}}\left( {n = 1,{\varvec{p}}} \right)$$

where ${\varvec{y}}_{{{\varvec{t}} + 1}}$ is a vector of length N (the number of grid cells within the study region) comprised of 0 s except for one element which is equal to 1, representing the chosen grid cell. The elements of the vector ${\varvec{p}}$ are the individual probabilities of moving to each grid cell, given by Eq. 1. Next, we partition ${\varvec{y}}_{{{\varvec{t}} + 1}}$ into two mutually exclusive sets: (a) ${\varvec{y}}_{t + 1}^{\left( 1 \right)}$ is the set which includes the chosen grid cell and a sample of other cells that were not chosen; and (b) ${\varvec{y}}_{t + 1}^{\left( 0 \right)}$ is the set with all the remaining grid cells, all of which were not chosen. Similarly, we also partition the probability vector ${\varvec{p}}$ into the corresponding vectors ${\varvec{p}}^{\left( 1 \right)}$ and ${\varvec{p}}^{\left( 0 \right)}$. Using basic properties of the multinomial distribution, the conditional distribution of ${\varvec{y}}_{t + 1}^{\left( 1 \right)}$ given ${\varvec{y}}_{t + 1}^{\left( 0 \right)}$ is given by:

3 $${\varvec{y}}_{t + 1}^{\left( 1 \right)} |{\varvec{y}}_{t + 1}^{\left( 0 \right)} \sim {\text{Multinom}}\left( {n = 1,\frac{{{\varvec{p}}^{\left( 1 \right)} }}{{1^{{\varvec{T}}} {\varvec{p}}^{\left( 1 \right)} }}} \right)$$

where $1^{{\varvec{T}}}$ is the transpose of a vector comprised of ones and $1^{{\varvec{T}}} {\varvec{p}}^{\left( 1 \right)}$ is just the sum of all probabilities within the vector ${\varvec{p}}^{\left( 1 \right)}$. This expression is useful when fitting the TEHS model (and when fitting other SSF models) because the challenging denominator in Eq. 1 disappears when calculating $\frac{{{\varvec{p}}^{\left( 1 \right)} }}{{1^{{\varvec{T}}} {\varvec{p}}^{\left( 1 \right)} }}$. We rely on this conditional likelihood (i.e., Eq. 3) instead of the likelihood in Eq. 2 to obtain unbiased parameter estimates.

It is important to note that this conditional likelihood is valid regardless of how ${\varvec{y}}_{{{\varvec{t}} + 1}}$ is partitioned (i.e., regardless of how “available sites” are selected). Therefore, one does not need to use samples from the empirical distributions of turning angles and step lengths to determine the available habitat, as is traditionally done in step selection models. We confirm that the approach used to select step lengths does not influence parameter estimates using simulations (see Additional file 2: Appendix 2). However, using the conditional likelihood in Eq. 3 instead of the original likelihood in Eq. 2 results in information loss, ultimately resulting in decreased precision of the parameter estimates. This reveals the important tradeoff between computational speed (afforded by selecting only a subset of the available pixels) and precision in parameter estimates.

### Empirical analysis: giant anteater case study

#### Movement data

**Fig. 3:**
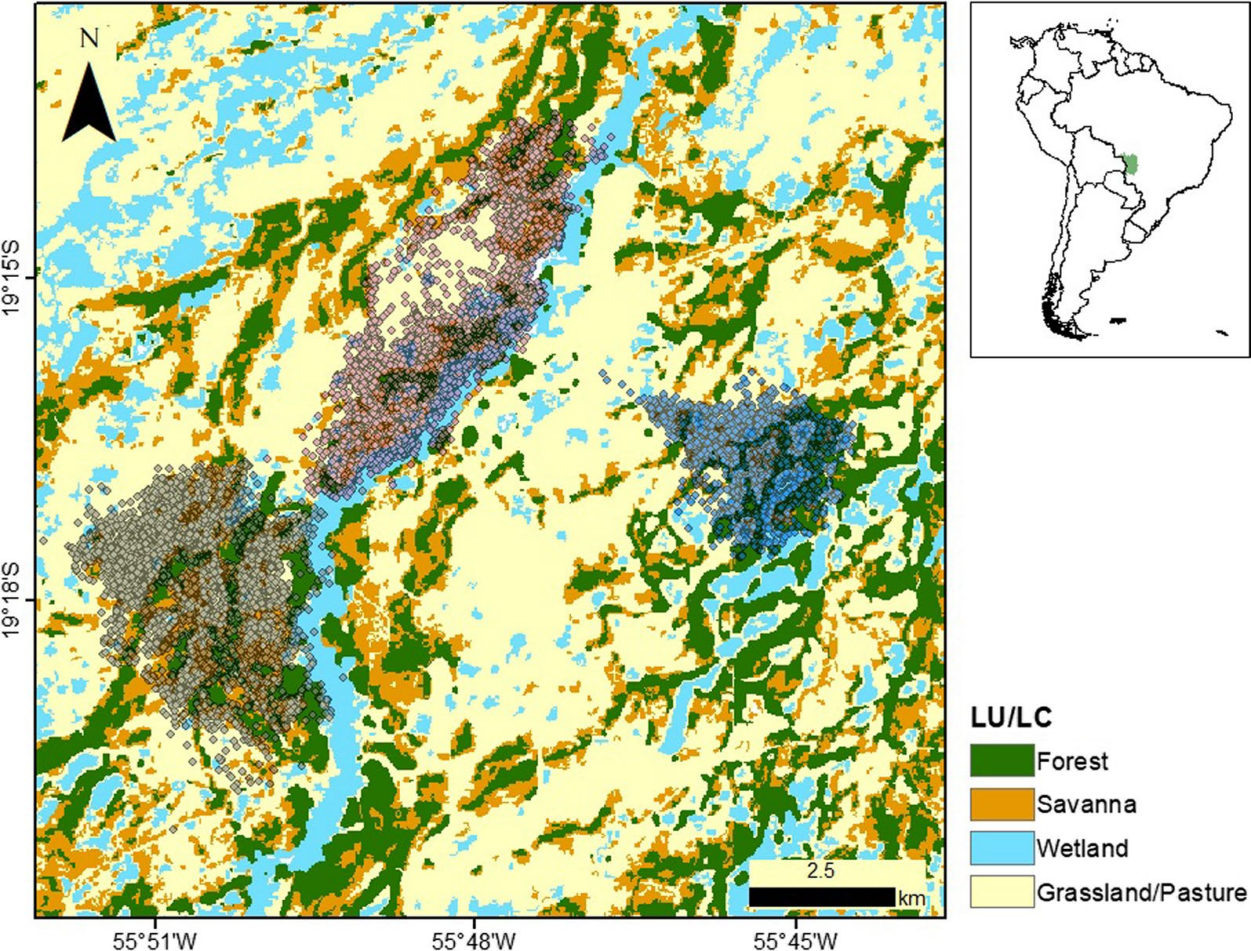
Land-use land-cover (LULC) classification of the study region for the year 2016 according to Mapbiomas (https://mapbiomas.org/en). Three individual giant anteaters (*Myrmecophaga tridactyla*) were monitored through GPS telemetry (semi-transparent circles) in the Brazilian Pantanal wetlands (green polygon on the inset)

The data were collected between 2013 and 2017 in a 350-km^2^ area in the Brazilian Pantanal (19° 16′ 60ʺ S, 55° 42′ 60ʺ W). The landscape is a mosaic of habitats that include forests, open grassland, pasture, savannas, and wetlands (Fig. 3). Historical mean temperature is 25.4 °C and climate is classified as semi-humid tropical (“Aw” in Köppen’s climate classification). Traditional extensive cattle ranching is practiced in the area, but overall anthropogenic impacts and threats are relatively low.

Anteaters were captured, immobilized, and sedated following the procedures described in Kluyber et al. [31]. Each individual was sexed, weighed, and equipped with a global positioning system (GPS, TGW-4570, Telonics) harness. None of the tracking devices exceeded 3% of the animals’ body mass. The GPS devices were programmed to record location points at 20 or 30-min intervals (depending on the animal). However, because some GPS fixes failed to be acquired, the time interval between fixes was sometimes greater than 20 or 30 min. As the time interval increases due to an increase in the number of failed GPS fixes, the assumption of a straight-line path becomes less reliable. For this reason, we removed observations for which the time interval was greater than 1 h and, whenever the time interval was greater than 35 min (to allow for some tolerance around the 30 min time interval), we allowed for increased variance in our time model by setting ${M}_{j}$ to one.

We also removed observations with speeds unlikely to be achieved by the species (> 0.33 m/s, approx. the 99th percentile of speed). Taken together, the removal of observations with very large time intervals or with unrealistic speeds resulted in the elimination of 2% of the data. Also, an important observation is that our time model is not defined when distances are exactly equal to zero. Therefore, whenever the distance between two GPS fixes was equal to zero (0.2% of our observations), we set distance to the smallest non-zero distance that was recorded (i.e., 1 m). The final movement dataset contained ~ 65,000 observations from three individuals (Table 1).

**Table 1:** Summary of the movement data used by the time and the habitat selection models. F and M stand for female and male, respectively

ID	Sex	Monitoring period		Duration (days)	Number of observations	
		Start	End		Time model	TEHS
Berenice	F	Oct 2015	Nov 2016	386	26,996	15,577
Brigite	F	Jul 2013	Jul 2014	340	11,233	7510
Fergus	M	Jul 2016	Aug 2017	407	27,010	13,115
Total				1133	65,239	36,202
Average				378	21,746	12,067

To characterize the habitat, we relied on Collection 5 of land-use land-cover classification (LULC) provided by Mapbiomas for the Pantanal ecosystem (https://mapbiomas.org/en) for each year between 2013 and 2017. Pixel size of this LULC product is 30 × 30 m. We also relied on hourly temperature data collected by the nearest automatic meteorological station of the National Institute of Meteorology of Brazil (INMET). Given that these temperature data occasionally exhibited abnormal temporal patterns (e.g., sudden drops followed by sudden increases of temperature) and there were some missing data, we decided to rely on the median temperature for each hour in each month and each year as a more robust measure of temperature that reflects both within day variation as well as seasonal variation.

#### Fitting the time model

For each step, defined as the straight line between two consecutive locations, we extracted the proportion of each LULC cover within a 30 m buffer of the path to account for GPS location and individual path uncertainty [56]. We combined the grasslands and pasture classes (hereafter grasslands), and then used it as the baseline LULC. As a result, we only include the proportion of forest, savanna, and wetland as covariates in our time model. Finally, to account for diel patterns in movement, we relied on cyclic cubic B splines to characterize how time taken to traverse a particular path depends on time of day, where the knots were set to 6 a.m., 12 p.m., and 6 p.m.

We fit this model in a Bayesian framework using JAGS [43]. Separate models were fit for each individual. Vague priors were adopted for $\gamma_{0} ,\gamma_{1} ,$ and $\beta_{0}$ whereas we used more conservative priors for the slope parameters $\beta_{p}$ (*p* = *1, …,P*):

$$\begin{aligned} & \gamma_{0} ,\gamma_{1} ,\beta_{0} \sim {\text{N}}\left( {0,10} \right),{\text{ and}} \\ & \beta_{p} \sim {\text{N}}\left( {0,1} \right)\quad {\text{for}}\quad p = 1, \ldots ,P. \\ \end{aligned}$$

A tutorial describing how to prepare the data and fit the time model is provided in Additional file 3: Appendix 3.

#### Fitting the habitat selection model

As described above, one does not have to rely on the empirical distributions of turning angles and step lengths to create the potential steps that the animal could have taken. Instead, we chose to create four potential steps of the same length as the observed step, one for each cardinal direction (i.e., east, west, north, and south from where the step started). Similar to the analysis for the time model, the proportion of each LULC class in the area surrounding each step was calculated by creating a 30-m buffer around the straight line that connects two consecutive locations and determining the proportion of pixels associated with each LULC class. Furthermore, although grasslands/pastures are present in the study region, we did not include this LULC class as covariate in the model because they act as the baseline LULC class. Finally, we removed observations with missing temperature data and steps for which LULC was identical for the observed and available steps (see sample size for each individual in Table 1). The reason for this last procedure is that there is no information on selection strength if the habitat information is the same for the observed and available steps because these cancel out when calculating the SSF ratio [37, 52].

We also fit this model in a Bayesian framework using JAGS [43]. Separate models were fit for each individual using the following conservative priors for the slope parameters:

$$\alpha_{p} \sim {\text{N}}\left( {0,1} \right)\quad {\text{for}}\quad p = 1, \ldots ,P.$$

A tutorial describing how to incorporate the time model results and fit the TEHS model is provided together with this article (Additional file 3: Appendix 3).

#### Connectivity implications

Recall that once the parameters of the TEHS model (i.e., the time model and the habitat selection model) have been estimated, they can be used to calculate the movement probabilities $q_{ij} = p\left( {P_{{t + {\Delta }t}} = j|{\Delta }t,P_{t} = i} \right)$ in Eq. 1 and create the permeability matrix **Q**, one of the key components of circuit theoretic connectivity analysis and SAMC. In this section, we examine the implications of the TEHS results in terms of characterizing permeability in heterogeneous and fragmented landscapes using SAMC.

To estimate the amount of flow of individuals through heterogeneous landscapes, the expected number of individuals in each pixel after *t* time steps was calculated as ${\text{N}}{{\varvec{\Psi}}}^{{\text{T}}} {\varvec{Q}}^{t}$, where T denotes the transpose operation and $N{{\varvec{\Psi}}}$ characterizes the initial distribution of individuals in each pixel of the landscape [17]. To illustrate these time-explicit calculations, we created a hypothetical landscape composed by a large wetland surrounded by grasslands with two patches of savanna and estimated the flow of individuals at different points in time. We assume that 100 individuals start at one savanna patch at the beginning of the simulation aiming to reach the other savanna patch. To set a savanna patch to be the destination, we selected a pixel $i^{*}$ at the center of this patch and we modified the **Q** matrix by setting $q_{{i^{*} j}} = 0$ for $i^{*} \ne j$ and $q_{{i^{*} i^{*} }} = 1$.

## Results

### Time model results

**Fig. 4:**
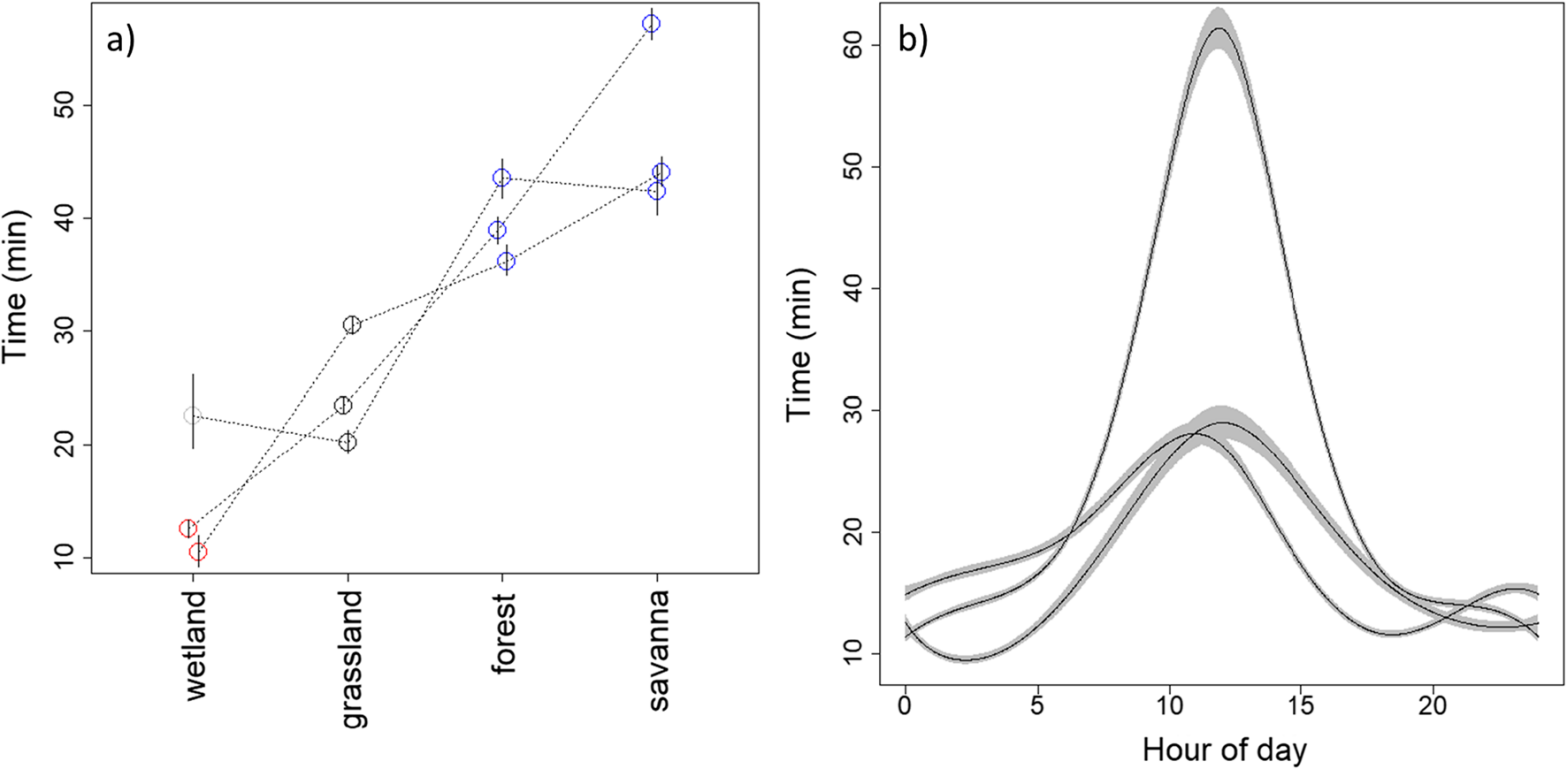
Results from the time model. **a** Estimated mean time (in min) taken to traverse 50 m in different types of habitat at 8 p.m. Each circle represents the result for a given animal and LULC category. Circles connected by the same line correspond to posterior median results from the same individual. Blue and red circles denote statistically positive (i.e., p($\beta$>0|D) > 0.975) and negative effects (i.e., p($\beta$<0|D) > 0.975), respectively, whereas grey circles show results that are not statistically discernible from grasslands (the baseline LULC class). In these equations, D denotes the dataset used to fit the model. Vertical lines are 95% credible intervals. **b** Estimated mean time required to traverse 50 m in the baseline LULC class (grasslands) throughout the day, showing that animals moved slower and were more likely to be inactive during the daytime. Each line corresponds to the posterior median for an individual animal and gray ribbons are the corresponding 95% pointwise credible intervals

The results for the time model applied to the simulated data showed that our model was able to estimate the true parameters well despite relying on an approximation where covariates are averaged along each step, even for landscapes that are more spatially heterogeneous (Additional file 1: Appendix 1). The results for the time model applied to the giant anteater data show that individuals consistently moved slower when traversing forests and savannas and faster when traversing wetlands, in comparison to the time taken to traverse grasslands (the baseline LULC) (Fig. 4a). Furthermore, based on our cyclic splines, we also find that giant anteaters tended to move much slower during daytime, from approximately 5 a.m. to 8 p.m., indicating that this species tends to have a nocturnal/crepuscular activity pattern (Fig. 4b). Finally, as expected, the $\gamma_{1}$ coefficients associated with the missed GPS fixes were consistently estimated to be negative, revealing that missed GPS fixes resulted in greater uncertainty in our time model (see Additional file 4: Appendix 4).

### Habitat selection function results

**Fig. 5:**
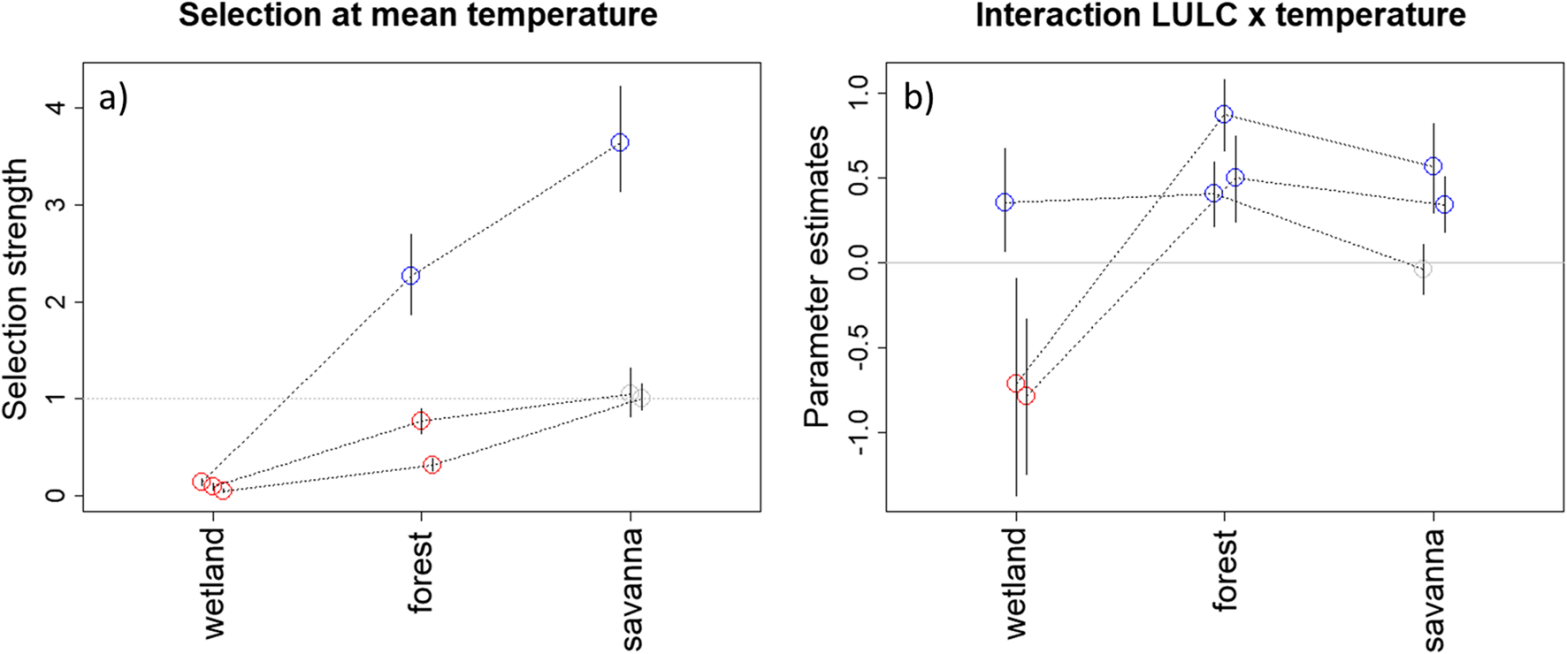
Estimates of (**a**) selection strength for different types of habitat at mean temperature (calculated as ${\text{exp}}\left({{\varvec{x}}}_{{\varvec{j}}}^{T}\boldsymbol{\alpha }\right)$) and (b) the effect of the interaction between LULC and temperature on selection strength. The horizontal grey line depicts the results for grasslands (the baseline LULC category) in panel A. Each circle represents the posterior median result for a given animal and LULC category and lines connect results from the same animal. Blue and red circles denote statistically positive (i.e., p($\alpha$>0|D) > 0.975) and negative effects (i.e., p($\alpha$<0|D) > 0.975), respectively, whereas grey circles indicate estimates that are not statistically discernible from zero. In these equations, D denotes the dataset used to fit the model. Vertical lines are 95% credible intervals

The results for the TEHS model, when applied to the simulated data, reveal that it can estimate well the habitat selection parameters, regardless of the number of available steps and the method used to choose these steps (Additional file 2: Appendix 2). Using the time model results described above, the TEHS model applied to the giant anteater data reveals that wetlands are avoided by all individuals relative to grasslands (the baseline LULC class) at the mean temperature of 25 °C (Fig. 5a). Although the selection for forests and savanna is ambiguous relative to grasslands, there is a consistent pattern of increased selection strength from wetlands to forests to savannas at mean temperature. Interestingly, the parameter estimates for the interaction between forest and temperature were consistently positive (Fig. 5b), indicating that selection strength for forests in relation to grasslands tends to increase with increasing temperatures. A similar pattern seems to hold for savanna, but generally the interaction is less strong and the result for one of the individuals was not statistically discernible from zero.

**Fig. 6:**
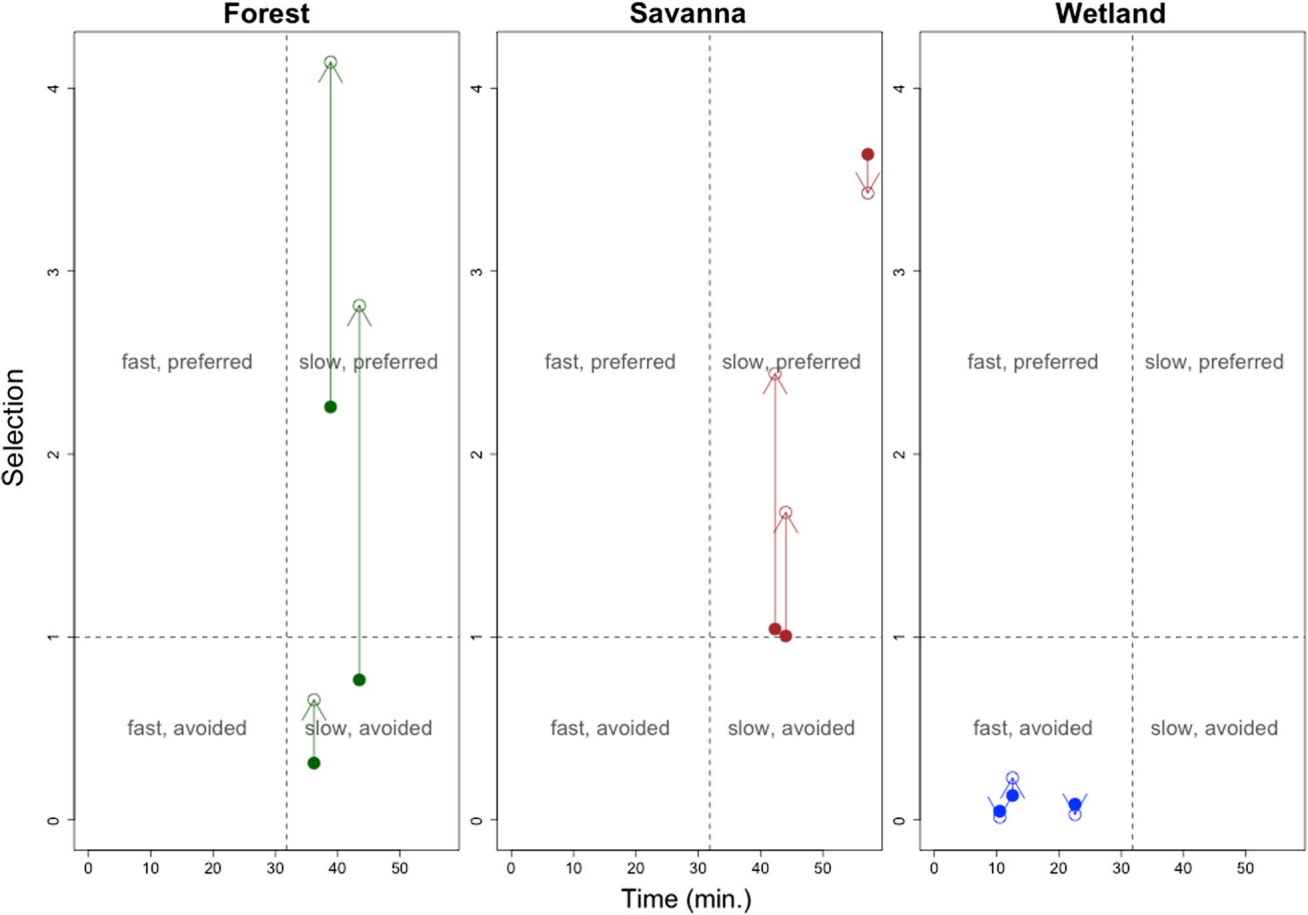
Characterization of landscape use by giant anteaters relative to time and selection strength (calculated as ${\text{exp}}\left({{\varvec{x}}}_{{\varvec{j}}}^{T}\boldsymbol{\alpha }\right)$) at two temperature scenarios. Each point represents the result for a particular individual in a given temperature scenario, and each panel shows the results for a LULC category. Estimated mean time value for each individual refers to the time taken to traverse 50 m of the LULC category at 8 p.m. Arrows indicate how selection strength is likely to change with increasing temperatures, connecting selection strength values at the mean temperature (25 °C; solid circles) and at 1.5 standard deviation above the mean (32 °C; open circles). The dashed vertical line represents the mean of the time estimates to traverse all LULC categories, whereas the dashed horizontal line represents the selection strength for grasslands (the baseline LULC category)

Combining the time model with the habitat selection model results enabled the characterization of landscape permeability and selection strength under different temperature scenarios. Figure 6 reveals that giant anteaters consistently move faster and avoid wetlands relative to grasslands, regardless of temperature (lower left quadrant). Furthermore, the individuals in our dataset generally selected for forests and savannas relative to grasslands, particularly at higher temperatures (upper right quadrant). These results suggest that animals rely on forests and savannas for slower behaviors (e.g., resting or foraging) and are more likely to increase their selection for these habitats as temperatures rise.

### Implications for connectivity

**Fig. 7:**
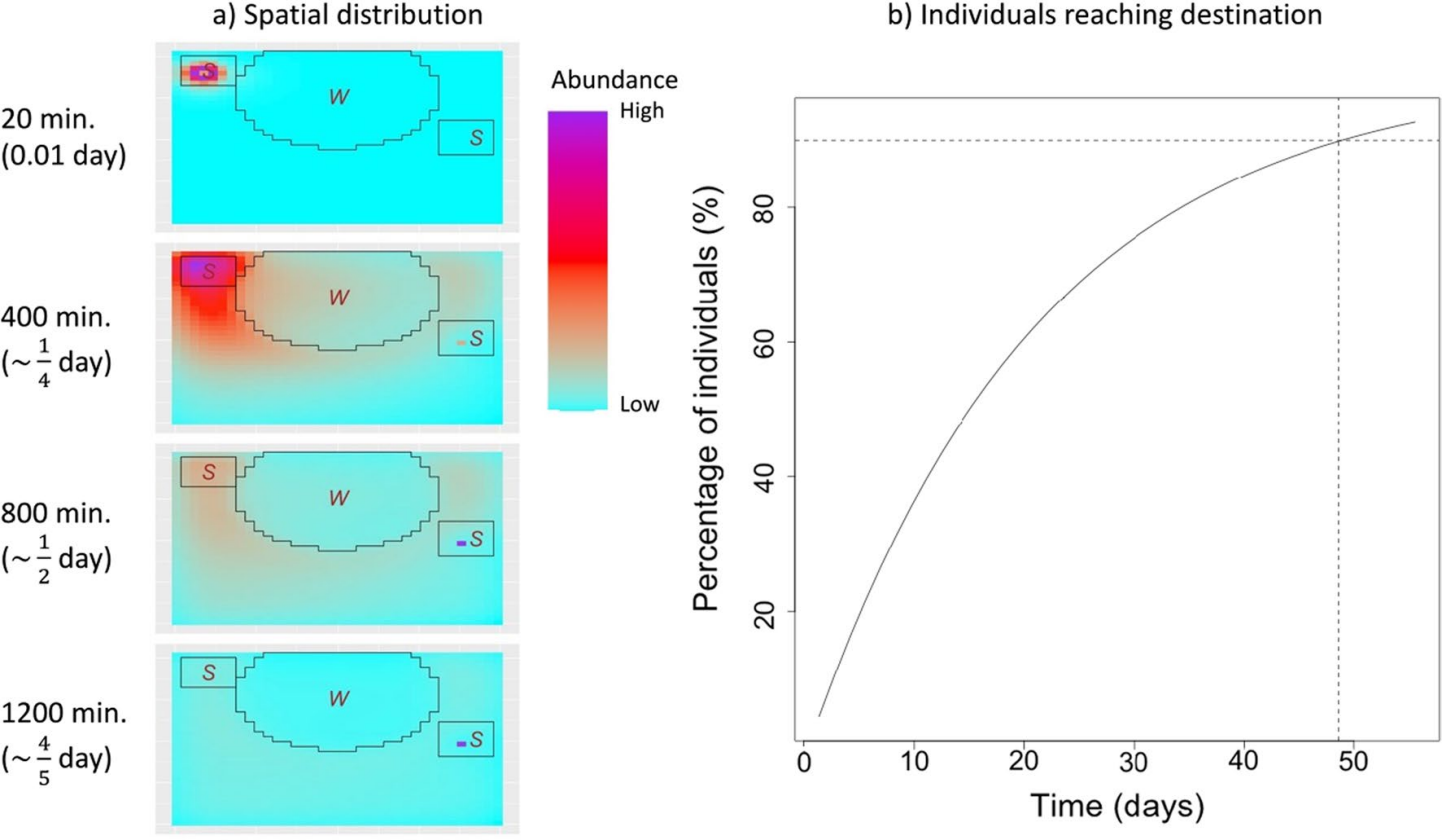
Connectivity implications of the inferred time and selection processes for a simulated landscape. This landscape consists of a wetland (ellipse with “W”) and patches of savanna (rectangles with “S”) within a grassland matrix (rest of the area). One hundred individuals start in the upper left savanna patch at time 0 and each panel in (**a**) shows the predicted abundance of individuals on each pixel after 1, 20, 40, and 60 time steps, where each time step corresponds to 20 min. The color gradient indicates predicted abundance of individuals, but note that the scale is not the same across different panels. Results in (**b**) show the estimated percentage of individuals in the destination patch as a function of time. Horizontal and vertical dashed lines highlight that approximately 49 days are required for 90% of the individuals to have reached the destination patch. All results are based on the estimated parameters for a single individual, assuming movement patterns at 8 p.m. and at mean temperature (25 °C)

Recall that the simulated landscape (Fig. 7) contains a large wetland (ellipse) surrounded by grasslands with two patches of savanna (rectangles) and that individuals start at the upper left patch (origin patch) and end up in the patch to the right of the wetland (destination patch). After parameterizing the SAMC permeability matrix with the TEHS estimates, our time-explicit predictions reveal that individuals do not use the shortest path to the destination patch because that would have required them to traverse the wetland, an avoided habitat type. Instead, these individuals tend to move around the wetland to eventually reach the destination patch (Fig. 7a). Importantly, our results suggest that approximately 49 days are required for 90% of the individuals to reach the destination patch (Fig. 7b).

## Discussion

In this article, we have proposed the time model which, when used together with a habitat selection function, yields the Time-Explicit Habitat Selection (TEHS) model. We have shown how these models can provide complementary information by explicitly distinguishing the drivers of time from the drivers of habitat selection in animal movement. Furthermore, we have shown how the TEHS model can be integrated into frameworks focused on estimating landscape connectivity, resulting in time-explicit predictions. Below we compare the TEHS model to previous habitat selection models and discuss our findings based on the giant anteater data from the Pantanal region.

### Comparison with previous modeling approaches

The time model proposed in this study is different from earlier approaches because it focuses on modeling time required to traverse a particular path (e.g., 7 min. to traverse 6 pixels; see Fig. 2) rather than distance, speed, or velocity. Although similar ecological insights are likely to be gained by modeling step length or speed, focusing on time is an important modeling choice for two reasons. First, many connectivity problems inherently require time-explicit solutions (e.g., if species can track a changing climate) and linking movement model results to a time-explicit framework (e.g., SAMC) requires a model for time (not distance, speed, or velocity) to be able to calculate the elements ${q}_{ij}=p\left({P}_{t+\Delta t}=j|\Delta t,{P}_{t}=i\right)$ in the permeability matrix **Q**. Second, assuming a gamma process and a straight-line assumption, this model enables inference on how characteristics of individual pixels influence time taken to traverse these pixels. On the other hand, speed (or velocity) at the step scale is not easily translated into speed (or velocity) at the pixel level, precluding the understanding of how speed (or velocity) relates to pixel-level characteristics.

The TEHS model contains a landscape-dependent kernel (i.e., the time model), which describes the probability of requiring a given amount of time to cross a heterogeneous landscape. As a result, this kernel can capture the fact that some landscape characteristics may facilitate faster movements while other characteristics may impede these movements. The TEHS model is most similar to the integrated step selection analysis (iSSA) described in Avgar et al. [4] but there are important differences. In iSSA, it is assumed that the probability of moving to a new location ${P}_{t+1}$ given the present location ${P}_{t}$ is given by: 4

where $\Omega$ is the study region, $w\left({P}_{t},{P}_{t+1}\right)$ is the habitat selection function, and $\phi \left({P}_{t+1}|{P}_{t}\right)$ accounts for movement constraints [4, 39]. The assumption that the probability of moving from one location to another can be expressed as being proportional to the multiplication of a selection function and a movement kernel (i.e., a separable model; [4]) is an important assumption of iSSA and other SSFs as well [39].

In the TEHS model, we rely on Bayes theorem to describe the probability of moving to a new location ${P}_{t+\Delta t}$ given the present location ${P}_{t}$:5

This expression is similar to the one in iSSA in that both $p\left(\Delta t|{P}_{t+\Delta t},{P}_{t}\right)$ and $\phi \left({P}_{t+1}|{P}_{t}\right)$ account for movement constraints and that $p\left({P}_{t+\Delta t}|{P}_{t}\right)$ and $w\left({P}_{t},{P}_{t+1}\right)$ are the habitat selection functions. However, despite the similarities in eqns. 4 and 5, we believe that our model is more principled because it relies on a mathematical fact (i.e., Bayes theorem, not to be confused with Bayesian statistics) instead of the separable assumption from iSSA [4], an assumption that may or may not be valid. Critically, it is because of Bayes theorem that we focus on modeling time taken to traverse the path instead of the more usual approach of modeling step lengths.

Finally, we note that standard statistical models typically adopt link functions to avoid obtaining non-sensical parameter values. For example, a logit link for the success probability in a logistic regression ensures that this probability is always between zero and one whereas a log link for $\lambda$ in a Poisson regression ensures that $\lambda$ (the mean of a Poisson distribution) is always positive. Unfortunately, because a conditional logistic regression is used to estimate the iSSA parameters, the estimated parameter values for the step length and turning angle distributions might be non-sensical (e.g., negative values for parameters of a Gamma distribution used for step lengths). This is exemplified in Additional file 5: Appendix 5. Although non-sensical values may or may not arise for any given dataset, the possibility that this might happen is an important limitation regarding how the iSSA parameters are currently estimated. Finally, the developers of iSSA noted that the “movement components of the iSSA are inherently ‘correlation-prone’ and are hence vulnerable to estimability issues” [4]. Our simulation results in Additional file 6: Appendix 6 and results based on the empirical data in Additional file 7: Appendix 7 corroborate this statement. On the other hand, the TEHS model and the adopted two-stage model fitting approach are able to avoid both of these problems.

### Giant anteater case study and connectivity implications

The time model applied to data from giant anteaters in the Pantanal region revealed that individuals tended to be most active between 8 p.m. and 5 a.m. Nocturnal behavior has been recorded for this species, especially on warmer days [10, 40]. Furthermore, we found that individuals tended to consistently move faster over wetlands, possibly because these environments are relatively poor in feeding resources when flooded and provide little vegetation cover, which increases predation risk. This hypothesis seems to be corroborated by the TEHS results, which revealed that wetlands were consistently avoided relative to all other land cover classes. In contrast, giant anteaters tend to move slowest over forests and savannas. This slower movement might be associated with not only increased biomechanical resistance offered by more vegetation, but also the fact that these habitats are used for foraging and resting [8, 20]. Indeed, the TEHS model showed increased selection strength for forests and savannas, particularly at higher temperatures. Previous studies suggest that forests may act as a thermal shelter for giant anteaters, not only when temperatures are high and above their thermoneutral zone, but also when temperatures are very low [10, 19]. One of the challenges of determining the effect of temperature on animal behavior is that it is highly correlated with the time of the day. In this study, we chose to incorporate hour of day in the time model, whereas temperature was included in the habitat selection function. However, a more complete understanding of the effect of temperature on individual behavior will require additional studies to better disentangle these processes.

By interpreting time and selection strength as distinct axes that govern animal behavior, we were able to characterize each LULC class in a biologically meaningful way (see Box 1) that can have important implications for conservation. For example, wetlands consistently fell in the “fast, avoided” quadrant, regardless of temperature. In contrast, we find that forests and savannas tend to fall in the “slow, selected” quadrant, particularly with higher temperatures. This suggests that while these habitats might not favor fast movement, they may be critical for the foraging and resting of giant anteaters, being good representatives of slow corridors [5]. Interestingly, none of our estimates fell in the “fast, selected” quadrant (upper left quadrant), which could potentially be the prime target when designing wildlife corridors [34]. We hypothesize that this might be due to the fact that all of the individuals in our dataset were resident individuals and dispersing individuals might have important differences in terms of speed and habitat selection when compared to resident individuals [6].

Similar to some recent work (e.g., [25, 45, 50]), this article contributes to the incipient literature that uses step-selection model results to inform connectivity analysis. More specifically, we show that the TEHS model results can be used to directly populate models for connectivity assessments rather than using resistance maps that require arbitrary decisions to relate measures of habitat quality (e.g., resource selection strength) to landscape resistance. Critically, using the TEHS model together with SAMC allows us to generate time-explicit predictions of dispersal patterns, something that many connectivity models cannot represent. We note, however, that our dispersal example has several limitations. First, this example is based on just one individual, not all three individuals in our dataset. Second, our results are based on the TEHS parameter estimates for a particular time of day and temperature. Similar to many connectivity analyses [33], our results do not account for individual or temporal variability (e.g., diel patterns and seasonal changes in activity level and temperature) but it has long been acknowledged in the literature that landscape connectivity is dynamic [53, 54]. Third, we do not take mortality risk or energetics into account even though this is a critical piece of information for more realistic connectivity analysis [17, 27]. Finally, it can be computationally challenging to scale up our connectivity analysis because we allow for transitions beyond the 8 nearest neighbors, resulting in a much denser (i.e., less sparse) matrix than most connectivity applications [16].

### Model limitations and future improvements

Our proposed models are better suited for shorter time intervals because we assume a linear path between GPS fixes. This linear path assumption is arguably the strongest assumption that our models make, and this assumption directly influences (a) the landscape covariate values that are used within the model; and (b) our estimate of the distance traversed. To take into account the fact that the exact path between two GPS fixes is unknown and therefore the environmental characteristics may not correspond to those in a straight line, we used a 30-m buffer around this straight line. One could also potentially use Brownian motion/diffusion to better characterize the environment [28]. This would be particularly useful when one or more GPS fixes are missing because, in these cases, a much larger area would have to be considered to properly characterize the environment. Alternatively, a potential path taken by the animal could be sampled after fitting a continuous-time model [26, 30, 48]. Finally, a third option would be to restrict the analysis of the time model to steps that occur in relatively homogeneous landscapes. In this situation, there is much less ambiguity regarding the characteristics of the environment that was traversed.

In relation to traversed distance, the assumption of a straight-line path almost certainly leads to an underestimate of this distance, but there are relatively limited options to circumvent this problem at the moment. Aside from sampling a potential path from a continuous time movement model [26, 30, 48], another option would be to use a dead-reckoning approach to better approximate the actual path taken by the individual [9, 41]. Unfortunately, this approach relies on specialized sensors (e.g., accelerometers and magnetometers) that are still relatively uncommon in the field, requires estimation of speed (e.g., as a function of dynamic body acceleration [DBA]), and the required calculations can be challenging to implement (but see [21]). Nevertheless, if and when dead-reckoning becomes a more commonly adopted method, the time model will still be useful and will yield more realistic results by not having to rely on a straight path assumption.

Another important limitation is that, similar to the distributions that are often used to model step length (e.g., gamma distribution) in Hidden Markov Models and iSSA [4], the time model assigns zero probability for distances that are equal to zero. This is not a problem from the perspective of model fitting because very few observations had distances exactly equal to zero. Indeed, even if the monitored individual is not moving, the distance between two GPS fixes is typically positive because of geolocation error. However, this characteristic can be problematic when inferring dispersal and connectivity patterns because it assumes that the monitored individual never stays in the same location for two consecutive time-steps. To circumvent this issue, the time model could be modified to include a two-stage process. In the first stage, the animal decides to either stay in the same location or move. If the animal decides to move, then the time model can be used to understand the time needed to traverse landscapes with different characteristics. Such a model would require distinguishing from the GPS tracking data when the animal is not moving from when the animal is exhibiting limited movement. Extending the time model to account for this two-stage process is an important area for future research. Finally, it is important to note that Eq. 1 does not explicitly represent turning angles. Modifying the TEHS model to enable the explicit representation of directional persistence is an important area for future research.

## Conclusions

Landscapes across the world are changing at ever increasing rates due to habitat degradation and loss associated with land use change. Connectivity analysis plays a central role in mitigating these impacts by identifying potential dispersal routes and corridors but better connecting these analyses with movement data is paramount to ensure the reliability of its results. Furthermore, time-explicit predictions of the flow of individuals through the landscape are critical to many connectivity problems but few modeling frameworks can generate such predictions. The methods proposed here can help characterize the ecological and functional roles of different habitat features, deepening our understanding of animal habitat selection patterns, and can improve how movement-based modeling results are incorporated into connectivity analysis, resulting in time-explicit landscape connectivity predictions.

### Supplementary Information

**Additional file 1. Appendix 1**. Simulations for the time model.

**Additional file 2. Appendix 2**. Simulations for the Time-Explicit Habitat Selection (TEHS) model.

**Additional file 3. Appendix 3**. Tutorial on preparing the data and fitting the models.

**Additional file 4. Appendix 4.** Results associated with $\gamma_{1}$ in the time model.

**Additional file 5. Appendix 5**. Nonsensical parameter estimates associated with the movement kernel within iSSA.

**Additional file 6. Appendix 6**. Parameter identifiability problems associated with the iSSA model.

**Additional file 7. Appendix 7**. Comparing iSSA to the TEHS model with a validation exercise.

## Data Availability

Movement data for the giant anteaters are available for visualization in Movebank (www.movebank.org with study name “Myrmecophaga tridactyla Pantanal”). Data download requests may be sent to the Center for Species Survival Brazil (SSC) from the International Union for Conservation of Nature (IUCN) (csebrasil.contato@gmail.com/fabiana@cpsg.org). The LULC class data are freely available at https://mapbiomas.org/en/download whereas the temperature data are available from the Brazilian National Institute of Meteorology (INMET; https://portal.inmet.gov.br/dadoshistoricos).

## Abbreviations

LULC: Land-use/land-cover
TEHS: Time-Explicit Habitat Selection model
iSSA: Integrate step selection analysis
GPS: Global positioning system
SAMC: Spatial absorbing Markov chain
JAGS: Just another Gibbs sampler
NDVI: Normalized difference vegetation index
INMET: National Institute of Meteorology of Brazil
DBA: Dynamic body acceleration
