# Supplementary material for: Bridging the gap between movement data and connectivity analysis using the Time-Explicit Habitat Selection (TEHS) model
Source: Mov Ecol. 2024 Mar 1;12:19. doi: 10.1186/s40462-024-00461-1 (PMC10908110; doi:10.1186/s40462-024-00461-1)
Supplement: Supplementary file 4 — Additional file 4. Appendix 4. Results associated with $\gamma_{1}$γ1 in the time model. [file 40462_2024_461_MOESM4_ESM.docx]

# Appendix 4. Results associated with $\gamma_{1}$ in the time model

Here we report the results for the parameter $\gamma_{1}$ from the time model applied to the giant anteater case study. Recall that this parameter quantifies if the time taken to traverse a particular area has greater uncertainty when one or more GPS fixes are missed. All of the 99% credible intervals for $\gamma_{1}$ did not overlap zero and the posterior medians were negative (Table S1), revealing that observations with missed GPS fixes tend to have greater uncertainty. This is expected given that there is less information regarding the actual path taken by the animal within that timeframe.

Table S1. Posterior summaries for $\gamma_{1}$.

| ID | Median | 99% credible interval | |
| --- | --- | --- | --- |
|  |  | 0.5% | 99.5% |
| Berenice | -0.84 | -1.01 | -0.66 |
| Brigite | -0.46 | -0.58 | -0.35 |
| Fergus | -0.76 | -1.02 | -0.54 |
