# Supplementary material for: Bridging the gap between movement data and connectivity analysis using the Time-Explicit Habitat Selection (TEHS) model
Source: Mov Ecol. 2024 Mar 1;12:19. doi: 10.1186/s40462-024-00461-1 (PMC10908110; doi:10.1186/s40462-024-00461-1)
Supplement: Supplementary file 5 — Additional file 5. Appendix 5. Nonsensical parameter estimates associated with the movement kernel within iSSA. [file 40462_2024_461_MOESM5_ESM.docx]

Appendix 5. Nonsensical parameter estimates associated with the movement kernel within iSSA.

Goal:

Using simulations, we show how fitting iSSA using a gamma distribution for its movement kernel can generate negative shape and scale parameters even if the data generating mechanism is based on positive parameter values.

Description of the iSSA model

Assume a gamma distribution for the movement kernel, given by:

$$p\left( y_{ij} \right)=\frac{\left( \frac{1}{q} \right)^{k}}{\Gamma\left( k \right)}y_{ij}^{k-1}exp(-\frac{1}{q}y_{ij})$$

where $y_{ij}$ is the step-length (i.e., the distance between locations i and j) and k and q are the shape and scale parameters respectively. To keep our example simple, we remove the resource selection function and the distribution for turning angles, enabling us to write the iSSA model as:

$$p\left( P_{t+\Delta t}=j|\Delta t,P_{t}=i \right)=\frac{\exp\left( -\frac{1}{q}y_{ij}+\left( k-1 \right)\log\left( y_{ij} \right) \right)}{\sum_{w} \exp\left( -\frac{1}{q}y_{iw}+\left( k-1 \right)\log\left( y_{iw} \right) \right)}$$

where $P_{t}$ and $P_{t+\Delta t}$ are the locations at time t and time $t+\Delta t$, respectively.

Let $-\frac{1}{q}=\beta_{1}$ and $k-1=\beta_{2}$. Furthermore, assume that $\beta_{1}$ and $\beta_{2}$ are linear functions of covariate $x_{ij}\in\left[ 0,1 \right]$ (e.g., the proportion of a LULC class along the path between locations i and j). In other words,

$$\beta_{1ij}=\theta_{1}+\theta_{2}x_{ij}$$

$$\beta_{2ij}=\theta_{3}+\theta_{4}x_{ij}$$

As a result, we can write our original expression as

$$p\left( P_{t+\Delta t}=j|\Delta t,P_{t}=i \right)=\frac{\exp\left( \left[ \theta_{1}+\theta_{2}x_{ij} \right]y_{ij}+\left[ \theta_{3}+\theta_{4}x_{ij} \right]\log\left( y_{ij} \right) \right)}{\sum_{w} \exp\left( \left[ \theta_{1}+\theta_{2}x_{iw} \right]y_{iw}+\left[ \theta_{3}+\theta_{4}x_{iw} \right]\log\left( y_{iw} \right) \right)}$$

In relation to our constraints, we know that $q>0$ and therefore $\beta_{1ij}=\theta_{1}+\theta_{2}x_{ij}<0$. Similarly, because $k>0$, this implies that $\beta_{2ij}=\theta_{3}+\theta_{4}x_{ij}>-1$. Unfortunately, iSSA does not contain any restrictions on the values that $\theta_{1},\theta_{2},\theta_{3},\theta_{4}$ can take to ensure that these constraints are satisfied.

In our example, because $x_{ij}\in\left[ 0,1 \right]$, this implies that $\theta_{1}<0$ and $\theta_{1}+\theta_{2}<0$. Furthermore, this also implies that $\theta_{3}>-1$ and $\theta_{3}+\theta_{4}>-1$. As a result, the natural constraints in the parameters of the gamma distribution imply the following constraints on iSSA model parameters:

$$\theta_{1}<0$$

$$\theta_{1}+\theta_{2}<0$$

$$\theta_{3}>-1$$

$$\theta_{3}+\theta_{4}>-1$$

Simulation approach for iSSA

We simulate data for iSSA by sampling from:

$$p\left( P_{t+\Delta t}=j|\Delta t,P_{t}=i \right)=\frac{\exp\left( \left[ \theta_{1}+\theta_{2}x_{ij} \right]y_{ij}+\left[ \theta_{3}+\theta_{4}x_{ij} \right]\log\left( y_{ij} \right) \right)}{\sum_{w} \exp\left( \left[ \theta_{1}+\theta_{2}x_{iw} \right]y_{iw}+\left[ \theta_{3}+\theta_{4}x_{iw} \right]\log\left( y_{iw} \right) \right)}$$

We created artificial landscapes containing 900 pixels, with the individual starting in the middle of these landscapes. We randomly drew the location chosen by the individual from a categorical distribution with probabilities governed by the iSSA probabilities. To satisfy the constraints on parameter values when simulating the data, we drew these parameters from the following distributions:

$$\theta_{1}\sim Unif\left( -1,0 \right)$$

$$\theta_{2}|\theta_{1}\sim Unif\left( -1,-\theta_{1} \right)$$

$$\theta_{3}\sim Unif\left( -1,1 \right)$$

$$\theta_{4}|\theta_{3}\sim Unif\left( -1-\theta_{3},1 \right)$$

On total, 100 simulated datasets were created.

Bayesian model fitting (4 available pixels)

To mimic the approach adopted for the TEHS model, we randomly chose 4 additional pixels that were available but that were not chosen. On total, we simulated 1,000 of these landscapes and thus each iSSA dataset for the Bayesian model contained (1 selected + 4 available pixels) x 1,000 landscapes = 5,000 observations. The iSSA model was fitted within a Bayesian framework using JAGS, using code that is similar to the one used for the TEHS model.

MLE model fitting (all available pixels)

An important concern with selecting just a subset of the unused available pixels is that this approach might not adequately estimate the denominator of iSSA (Michelot et al. 2023). To show that our results are not related to this concern, we created another set of simulations in which we kept all 900 pixels in each landscape in our dataset. By assuming that space is discrete, this approach allows us to calculate the iSSA probability exactly (i.e., there is no need to approximate the denominator). Because this approach generates a very large dataset, we simulated only 200 landscapes for each dataset, resulting in 900 pixels x 200 landscapes = 180,000 observations. We fit ISSA using the function “clogit” of the R package “survival” (Therneau 2023). The 95% confidence intervals for $\theta_{1}+\theta_{2}$ and $\theta_{3}+\theta_{4}$ were estimated as $\theta_{1}+\theta_{2}\pm1.96\sqrt{Var\left( \theta_{1} \right)+Var\left( \theta_{2} \right)+2Cov\left( \theta_{1},\theta_{2} \right)}$ and $\theta_{3}+\theta_{4}\pm1.96\sqrt{Var\left( \theta_{3} \right)+Var\left( \theta_{4} \right)+2Cov\left( \theta_{3},\theta_{4} \right)}$, respectively.

Results for the Bayesian iSSA with 4 available pixels

Our results reveal that the model parameters were well estimated by the Bayesian iSSA, with a comparison between the estimated and the true parameters generally falling along the 1:1 line (Fig. S1). However, despite the fact that the simulations were created with parameter values that satisfied the constraints imposed by the gamma distribution assumption for step-lengths, some parameter estimates violated these constraints (red circles in Fig. S1). Furthermore, the resulting posterior distributions often placed considerable weight on nonsensical parameter values, as indicated by some of the 95% credible intervals violating these parameter constraints (red vertical lines in Fig. S1). The reason for this is because the iSSA, as currently formulated, places no restriction on parameter values.


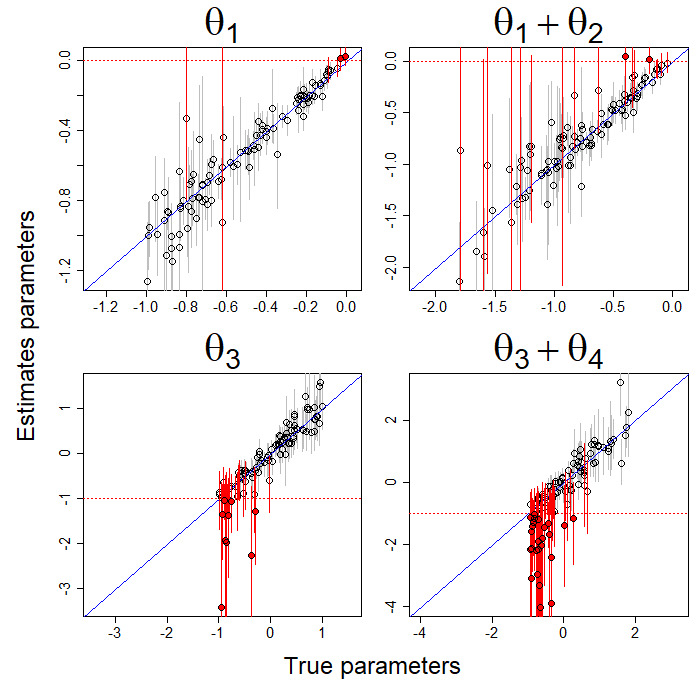


Fig. S1. Comparison of the true and estimated parameter values based on the Bayesian iSSA and 4 available pixels. A 1:1 line (blue diagonal line) was added for reference. Dotted horizontal red lines represent the constraint value for each parameter or combination of parameters. Each circle represents the result for one of the simulated data sets. Red and black circles are results that violate and that do not violate the constraint, respectively. The 95% credible intervals are depicted in red and grey when they overlap and do not overlap the constraint value, respectively.

Results for the MLE iSSA with all available pixels

Our results from the MLE iSSA are similar to those from the Bayesian iSSA. For example, we find that the model parameters were also well estimated by the MLE iSSA (Fig. S2). As expected, there was less uncertainty in parameter estimates when compared to the results in Fig. S1 because all available pixels were used. However, regardless of the fact that all available pixels were used, some parameter estimates (red circles in Fig. S2) and 95% confidence intervals (vertical red lines in Fig. S2) still violated the constraints required to ensure that the gamma parameters were positive.


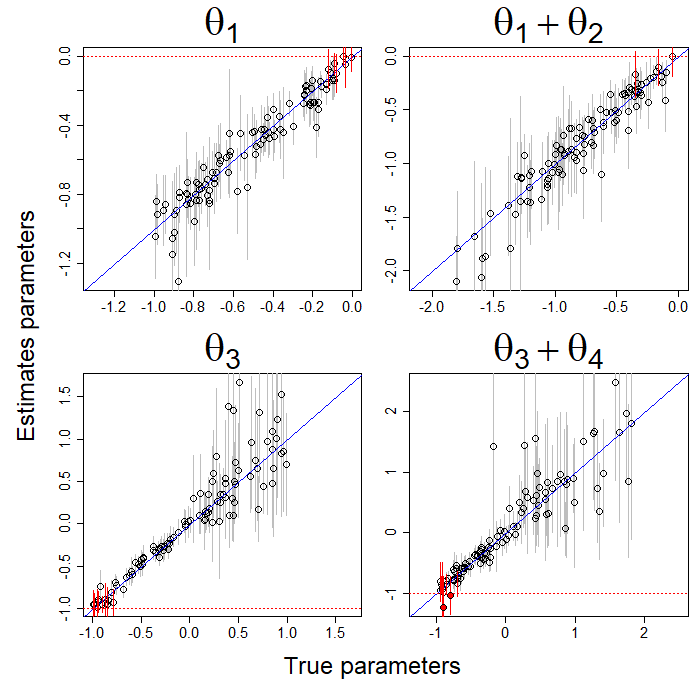


Fig. S2. Comparison of the true and estimated parameter values based on the MLE iSSA with all available pixels. A 1:1 line (blue diagonal line) was added for reference. Dotted horizontal red lines represent the constraint value for each parameter/set of parameters. Each circle represents the result for one of the simulated data sets. Red and black circles are results that violate and that do not violate the constraint, respectively. The 95% confidence intervals are depicted in red and grey when they overlap and do not overlap the constraint value, respectively.

In short, regardless of how iSSA is fit (MLE or Bayesian framework) and if only 4 or all available pixels are included in the simulated data, iSSA has the potential to generate parameter estimates that are nonsensical such as negative parameter values for the gamma distribution.

References

Michelot, T., N. J. Klappstein, J. R. Potts, and J. Fieberg. 2023. Understanding step selection analysis through numerical integration. Methods in Ecology and Evolution:1-12.

Therneau, T. M. 2023. A Package for Survival Analysis in R. <https://CRAN.R-project.org/package=survival:> <https://CRAN.R-project.org/package=survival>
