# Supplementary material for: Bridging the gap between movement data and connectivity analysis using the Time-Explicit Habitat Selection (TEHS) model
Source: Mov Ecol. 2024 Mar 1;12:19. doi: 10.1186/s40462-024-00461-1 (PMC10908110; doi:10.1186/s40462-024-00461-1)
Supplement: Supplementary file 6 — Additional file 6. Appendix 6. Parameter identifiability problems associated with the iSSA model. [file 40462_2024_461_MOESM6_ESM.docx]

Appendix 6. Parameter identifiability problems associated with the iSSA model.

Goal:

Using simulations, we show that fitting iSSA using a gamma distribution for its movement kernel can be challenging given the high correlation induced by including step-length, log(step-length), and their interaction with other covariates.

Description of the iSSA model

To simulate data for iSSA, we assume a gamma distribution for step-lengths. Furthermore, we assume that both parameters of the gamma distribution (shape and scale) are functions of two covariates $\bar{x}_{1ij}$ and $\bar{x}_{2ij}$. Similarly, we assume that selection is also a function of these two covariates. Notice that we do not include a distribution for turning angles. This model is given by:

$$p\left( P_{t+\Delta t}=j|\Delta t,P_{t}=i \right)=\frac{\exp\left( \beta_{0}\bar{x}_{1ij}+\beta_{1}\bar{x}_{2ij}+\beta_{2}y_{ij}+\beta_{3}\log\left( y_{ij} \right)+\beta_{4}\bar{x}_{1ij}\log\left( y_{ij} \right)+\beta_{5}\bar{x}_{2ij}\log\left( y_{ij} \right)+\beta_{6}\bar{x}_{1ij}y_{ij}+\beta_{7}\bar{x}_{2ij}y_{ij} \right)}{\sum_{k} \exp\left( \beta_{0}\bar{x}_{1ik}+\beta_{1}\bar{x}_{2ik}+\beta_{2}y_{ik}+\beta_{3}\log\left( y_{ik} \right)+\beta_{4}\bar{x}_{1ik}\log\left( y_{ik} \right)+\beta_{5}\bar{x}_{2ik}\log\left( y_{ik} \right)+\beta_{6}\bar{x}_{1ik}y_{ik}+\beta_{7}\bar{x}_{2ik}y_{ik} \right)}$$

where $y_{ij}$ is the distance between pixel i and pixel j (i.e., step-length).

We created artificial landscapes containing 900 pixels, with the individual starting in the middle of these landscapes. We randomly drew the location chosen by the individual from a categorical distribution with probabilities governed by the iSSA probabilities. Finally, we kept the chosen pixel and randomly chose 4 additional pixels that were available but that were not chosen. On total, we simulated 1,000 of these landscapes and thus each iSSA dataset contained (1 selected + 4 available pixels) x 1,000 landscapes = 5,000 observations.

In each landscape, we assumed that there were 3 types of land-use/land-cover (LULC) classes. These landscapes had a random spatial distribution of these classes except for some areas that were dominated by a given class, representing a certain degree of patchiness in the landscape. Fig. S1 shows an example of one of these simulated landscapes. The covariates $\bar{x}_{1ij}$ and $\bar{x}_{2ij}$ consisted of the proportions of LULC classes 1 and 2, respectively, in the path from grid cell i to grid cell j.


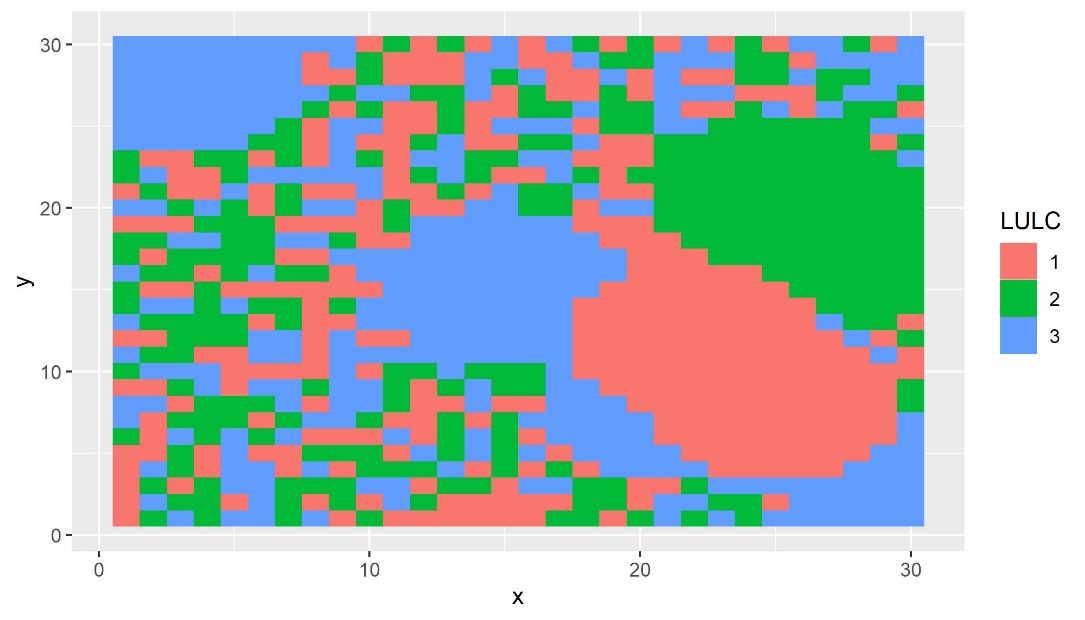


Fig. S1. Example of the spatial distribution of the LULC classes in the simulated landscapes

On total, 100 simulated datasets were created. Parameters $\beta_{0},\ldots,\beta_{7}$ were randomly drawn from a uniform distribution between -1 and 1. The iSSA model was fitted both in a Bayesian framework (using JAGS, with code that is similar to the one used for the TEHS model) and in a maximum likelihood framework (using the function “clogit” within the R package “survival”; Therneau 2023).

Results for iSSA

Our results using a Bayesian framework reveal that some of the iSSA model parameters were well estimated, with a comparison between the estimated and the true parameters generally falling along the 1:1 line, whereas other parameters were less well estimated (Fig. S2). Importantly, we find that 22% of the models fitted to the 100 simulated datasets had at least one parameter that did not converge (i.e., convergence statistic $\hat{R}$>1.1). Table S1 describes the proportion of times that each parameter had $\hat{R}$>1.1.


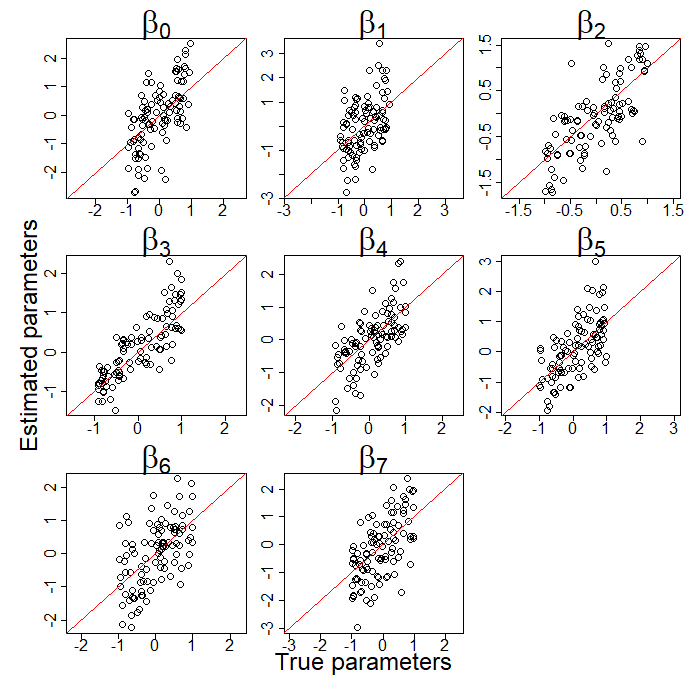


Fig. S2. Comparison of the estimated and true parameter values for each iSSA parameter. Parameters were estimated in a Bayesian framework. A 1:1 line was added for reference (diagonal red line). Each circle represents the result based on one of the 100 simulated datasets.

| Parameter | Proportion with lack of convergence |
| --- | --- |
| $\beta_{0}$ | 0.13 |
| $\beta_{1}$ | 0.13 |
| $\beta_{2}$ | 0.18 |
| $\beta_{3}$ | 0.14 |
| $\beta_{4}$ | 0.09 |
| $\beta_{5}$ | 0.09 |
| $\beta_{6}$ | 0.13 |
| $\beta_{7}$ | 0.13 |

Table S1. Proportion of times (out of 100 Bayesian models) that each parameter did not converge (i.e., convergence statistic $\hat{R}$>1.1).

We note that the correlation between $y_{ij}$ and $\log\left( y_{ij} \right)$ is problematic irrespective of the framework used to fit the model (i.e., Bayesian or MLE). For example, when these data are fitted in a maximum likelihood framework, we find a similar result to those from the Bayesian model when comparing the estimated and the true parameter values (Fig. S3). More critically however, we observe 95% confidence intervals with widths in the order of tens to hundreds, which are very large given that the true parameter values varied from -1 and 1 (Table S3).


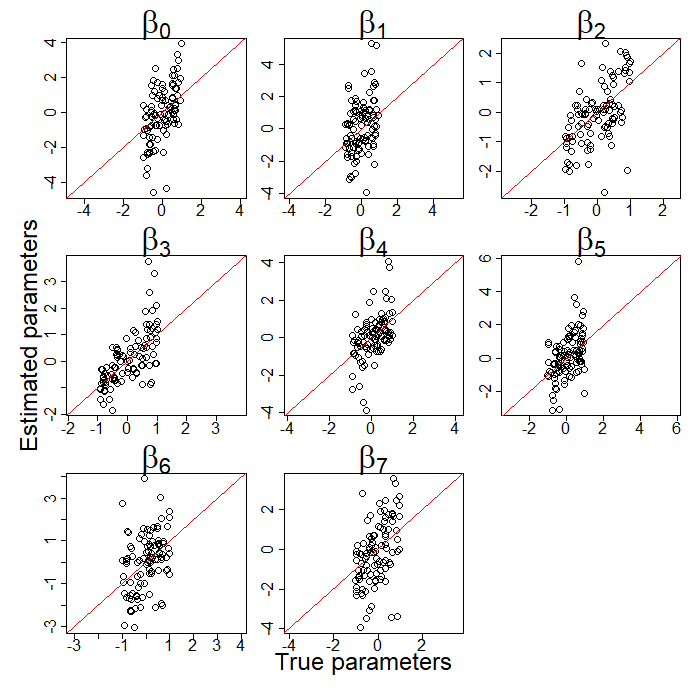


Fig. S3. Comparison of the estimated and true parameter values for each iSSA parameter. Parameters were estimated in a MLE framework using the R package “survival” (Therneau 2023). A 1:1 line was added for reference (diagonal red line). Each circle represents the result based on one of the 100 simulated datasets.

| Parameter | Mean width of 95% confidence intervals |
| --- | --- |
| $\beta_{0}$ | 48 |
| $\beta_{1}$ | 203 |
| $\beta_{2}$ | 6 |
| $\beta_{3}$ | 16 |
| $\beta_{4}$ | 83 |
| $\beta_{5}$ | 309 |
| $\beta_{6}$ | 41 |
| $\beta_{7}$ | 43 |

Table S2. Mean width of 95% confidence intervals for each parameter based on 4 available steps and 100 simulated datasets. Parameters were estimated in a MLE framework using the R package “survival” (Therneau 2023).

We also investigated if results would be different had we included all available pixels in the landscape as part of our datasets. In this case, the iSSA’s probabilities can be calculated exactly. However, because the resulting dataset would be very large, we simulated only 200 of these landscapes and thus each iSSA dataset contained 900 pixels x 200 landscapes = 180,000 observations. We find that, although shorter 95% confidence intervals arise when all available pixels are included in the simulated datasets, these intervals are still large relative to the size of the parameters that are being estimated (Table S3).

| Parameter | Mean width of 95% confidence intervals |
| --- | --- |
| $\beta_{0}$ | 10 |
| $\beta_{1}$ | 10 |
| $\beta_{2}$ | 6 |
| $\beta_{3}$ | 5 |
| $\beta_{4}$ | 7 |
| $\beta_{5}$ | 8 |
| $\beta_{6}$ | 9 |
| $\beta_{7}$ | 9 |

Table S3. Mean width of 95% confidence intervals for each parameter based on 100 simulated datasets containing all available pixels. Parameters were estimated in a MLE framework using the R package “survival” (Therneau 2023).

It is important to note that these large 95% confidence intervals are symptoms of multicollinearity problems. Indeed, we find very high correlation between the parameters estimated by iSSA (Table S4). This is a direct result of highly correlated covariates. For example, although the covariates $\bar{x}_{1ij}$ and $\bar{x}_{2ij}$ were not highly correlated (median correlation of -0.49), step-length and log(step-length) were strongly correlated (median correlation of 0.95).

|  | $\beta_{0}$ | $\beta_{1}$ | $\beta_{2}$ | $\beta_{3}$ | $\beta_{4}$ | $\beta_{5}$ | $\beta_{6}$ | $\beta_{7}$ |
| --- | --- | --- | --- | --- | --- | --- | --- | --- |
| $\beta_{0}$ | 1.00 |  |  |  |  |  |  |  |
| $\beta_{1}$ | 0.48 | 1.00 |  |  |  |  |  |  |
| $\beta_{2}$ | 0.77 | 0.77 | 1.00 |  |  |  |  |  |
| $\beta_{3}$ | -0.73 | -0.72 | **-0.94** | 1.00 |  |  |  |  |
| $\beta_{4}$ | **0.93** | 0.45 | 0.73 | -0.77 | 1.00 |  |  |  |
| $\beta_{5}$ | 0.45 | **0.94** | 0.74 | -0.77 | 0.47 | 1.00 |  |  |
| $\beta_{6}$ | **-0.98** | -0.47 | -0.79 | 0.73 | -**0.93** | -0.44 | 1.00 |  |
| $\beta_{7}$ | -0.48 | **-0.99** | -0.78 | 0.72 | -0.45 | **-0.94** | 0.48 | 1.00 |

Table S4. Median correlation between parameters of iSSA. Median was calculated based on the pairwise correlation between parameters over all 100 simulated datasets. Correlation between parameters were estimated in a MLE framework using the R package “survival” (Therneau 2023). Correlations that are greater in magnitude than 0.9 are emphasized in bold (except for the diagonal elements).

References

Therneau, T. M. 2023. A Package for Survival Analysis in R. <https://CRAN.R-project.org/package=survival>
