# Supplementary material for: Bridging the gap between movement data and connectivity analysis using the Time-Explicit Habitat Selection (TEHS) model
Source: Mov Ecol. 2024 Mar 1;12:19. doi: 10.1186/s40462-024-00461-1 (PMC10908110; doi:10.1186/s40462-024-00461-1)
Supplement: Supplementary file 7 — Additional file 7. Appendix 7. Comparing iSSA to the TEHS model with a validation exercise. [file 40462_2024_461_MOESM7_ESM.docx]

Appendix 7. Comparing iSSA to the TEHS model with a validation exercise

**Approach**

We performed a validation exercise to compare iSSA and the TEHS model. In this exercise, 10% of the data for each animal was randomly selected as validation data and the remaining 90% was used to train the models. We focused solely on LULC covariates (i.e., proportion of forest, savanna, and wetland) to keep the model simple (i.e., we dropped temperature and the time spline covariates).

For iSSA, we assumed a gamma distribution for the movement kernel and we assumed that this distribution was potentially influenced by the LULC covariates. For the TEHS model, both the time model and the habitat selection function were potentially influenced by these LULC covariates. We used the same set of 4 available steps for both models and fitted these models within a Bayesian framework using JAGS.

To determine if these models had estimability/identifiability problems, we calculated the correlation in posterior samples for the model parameters. Because we had data on three individuals, we summarized this information by calculating the median correlation for each pairwise correlation. We also assessed if iSSA was estimating parameter values that implied nonsensical parameters for the Gamma distribution used in the movement kernel. Finally, we quantified how well each model was able to predict the observed step for the validation data by calculating how often the observed step had a predicted probability equal to or higher than all the other available steps.

**Results**

We find that the median correlation in posterior samples of the TEHS model parameters were relatively low in magnitude, ranging from -0.67 to 0.30 for the time model (Table 1) and 0.14 to 0.27 for the habitat selection function (Table 2).

|  | Intercept | Forest | Savanna | Wetland |
| --- | --- | --- | --- | --- |
| Intercept | 1.00 |  |  |  |
| Forest | -0.60 | 1.00 |  |  |
| Savanna | -0.67 | 0.30 | 1.00 |  |
| Wetland | -0.31 | 0.17 | 0.23 | 1.00 |

Table 1. Median of the correlation in posterior samples of the parameters in the time model

|  | Forest | Savanna | Wetland |
| --- | --- | --- | --- |
| Forest | 1.00 |  |  |
| Savanna | 0.27 | 1.00 |  |
| Wetland | 0.24 | 0.14 | 1.00 |

Table 2. Median of the correlation in posterior samples of the parameters in the habitat selection function of the TEHS model.

On the other hand, the median correlation for the iSSA model parameters were very large in magnitude, ranging from -0.97 to 0.74 (Table 3), suggesting important estimability/identifiability problems.

|  | SL | Log(SL) | Forest | Savanna | Wetland | Forest*  SL | Sav.*  SL | Wet.*  SL | For.*  log(SL) | Sav.*  log(SL) | Wet.*  log(SL) |
| --- | --- | --- | --- | --- | --- | --- | --- | --- | --- | --- | --- |
| SL | 1.00 |  |  |  |  |  |  |  |  |  |  |
| Log(SL) | -0.07 | 1.00 |  |  |  |  |  |  |  |  |  |
| Forest | -0.01 | 0.01 | 1.00 |  |  |  |  |  |  |  |  |
| Savanna | 0.01 | 0.01 | 0.47 | 1.00 |  |  |  |  |  |  |  |
| Wetland | 0.01 | -0.02 | 0.09 | 0.09 | 1.00 |  |  |  |  |  |  |
| Forest*SL | 0.01 | 0.00 | 0.73 | 0.31 | 0.09 | 1.00 |  |  |  |  |  |
| Savanna*SL | 0.00 | 0.01 | 0.26 | 0.74 | 0.05 | 0.27 | 1.00 |  |  |  |  |
| Wetland*SL | 0.01 | 0.02 | 0.11 | 0.11 | 0.69 | 0.23 | 0.18 | 1.00 |  |  |  |
| Forest*log(SL) | 0.00 | -0.01 | **-0.96** | -0.42 | -0.09 | -0.87 | -0.28 | -0.17 | 1.00 |  |  |
| Savanna*log(SL) | -0.01 | -0.01 | -0.43 | **-0.96** | -0.08 | -0.33 | -0.88 | -0.14 | 0.42 | 1.00 |  |
| Wetland*log(SL) | -0.01 | 0.00 | -0.11 | -0.10 | **-0.97** | -0.13 | -0.10 | -0.85 | 0.13 | 0.11 | 1.00 |

Table 3. Median of the correlation in posterior samples of the iSSA parameters. Values greater in magnitude than 0.9 are highlighted in bold (except for the diagonal elements). SL stands for step-length.

In relation to nonsensical parameter values, we find that iSSA implicitly estimated negative values for the Gamma distribution parameters for a relatively large proportion of the observations in the training dataset for the giant-anteaters Brigite and Fergus but not for Berenice (Table 4).

| ID | Proportion of observations |
| --- | --- |
| Berenice | 0.00 |
| Brigite | 0.12 |
| Fergus | 0.14 |

Table 4. Proportion of observations in the training dataset for which iSSA implicitly estimated negative parameter values for the gamma distribution.

In relation to predictive skill, we find that iSSA and the TEHS model had similar predictive skill, with the TEHS model slightly outperforming iSSA for two individuals and underperforming for one individual (Table 5). These results illustrate that TEHS can avoid the parameter identifiability and nonsensical parameter values problems of iSSA while having similar predictive performance.

| ID | iSSA | TEHS |
| --- | --- | --- |
| Berenice | 0.34 | **0.36** |
| Brigite | **0.36** | 0.33 |
| Fergus | 0.36 | **0.37** |

Table 5. Proportion of observations for which the observed step had an equal or higher predicted probability than the available steps for the validation dataset. The best result for each individual giant anteater is highlighted in bold.
